# Targeting the CK1α/CBX4 axis for metastasis in osteosarcoma

**DOI:** 10.1038/s41467-020-14870-4

**Published:** 2020-02-28

**Authors:** Xin Wang, Ge Qin, Xiaoting Liang, Wen Wang, Zhuo Wang, Dan Liao, Li Zhong, Ruhua Zhang, Yi-Xin Zeng, Yuanzhong Wu, Tiebang Kang

**Affiliations:** 1 State Key Laboratory of Oncology in South China, Collaborative Innovation Center for Cancer Medicine, Sun Yat-sen University Cancer Center, Guangzhou, China; 2 The Sixth Affiliated Hospital, Sun Yat-sen University, Guangzhou, China; 3 Department of Abdominal Oncology, The Cancer Center of the Fifth Affiliated Hospital of Sun Yat-sen University, Zhuhai, Guangdong China; 4 Department of Pathology, The First Affiliated Hospital, Sun Yat-sen University, Guangzhou, China

**Keywords:** Metastasis, Sarcoma

## Abstract

Osteosarcoma, an aggressive malignant cancer, has a high lung metastasis rate and lacks therapeutic target. Here, we reported that chromobox homolog 4 (CBX4) was overexpressed in osteosarcoma cell lines and tissues. CBX4 promoted metastasis by transcriptionally up-regulating *Runx2* via the recruitment of GCN5 to the *Runx2* promoter. The phosphorylation of CBX4 at T437 by casein kinase 1α (CK1α) facilitated its ubiquitination at both K178 and K280 and subsequent degradation by CHIP, and this phosphorylation of CBX4 could be reduced by TNFα. Consistently, CK1α suppressed cell migration and invasion through inhibition of CBX4. There was a reverse correlation between CK1α and CBX4 in osteosarcoma tissues, and CK1α was a valuable marker to predict clinical outcomes in osteosarcoma patients with metastasis. Pyrvinium pamoate (PP) as a selective activator of CK1α could inhibit osteosarcoma metastasis via the CK1α/CBX4 axis. Our findings indicate that targeting the CK1α/CBX4 axis may benefit osteosarcoma patients with metastasis.

## Abstract (web summary)

Osteosarcoma is an aggressive tumour and little is known the mechanisms underpinning its highly metastatic nature. Here, the authors highlight a role for the CK1α/CBX4 axis in driving metastasis, suggesting that this pathway might be targeted for therapeutic benefit.

## Introduction

Osteosarcoma is a primary malignant bone tumor that most commonly affects children, adolescents, and young adults^1,2^. Approximately 15–20% of patients have clinically detectable metastases at diagnosis. More than 85% of metastatic disease occurs in lungs, the most common site of metastasis, whereas bone is the second most common site of distant disease^1,3^. The survival of patients with metastatic or relapsed osteosarcoma has remained virtually unchanged over the past 30 years, with an overall 5-year survival rate of approximately 20%^4,5^. Therefore, the identification of strategies for osteosarcoma patients with metastatic or relapsed disease is necessary and urgent. Consequently, a better understanding of the molecular mechanisms of osteosarcoma tumorigenesis and identification of therapeutic targets for osteosarcoma are urgent research objectives.

CBX4 is one of the CBX protein family, including CBX2, 4, 6, 7, and 8, which has been reported to join in the polycomb repressive complex 1 (PRC1) and characterized as a transcriptional repressor^6^. CBX4 is also a special chromobox protein because it is a SUMO E3 ligase^7,8^, and it can act as both oncogene and tumor suppressor depending on the cell type as well as its interacting partners. For instance, CBX4 functions as a tumor suppressor by inhibiting the c-myc expression and cellular transformation^9^; CBX4 connects with E2F and Rb to repress the transcription of both *cyclin A* and *cdc2*, which consequently reduce cell proliferation, this function is involved in its PRC1 complex^10^; and CBX4 positively regulates proliferation, angiogenesis, and cancer metastasis in hepatocellular carcinoma (HCC), which is dependent on in its sumoylating E3 activity^6,11^. We have recently shown that CBX4 impairs colorectal carcinoma (CRC) metastasis by inhibiting *Runx2* transcription via recruiting HDAC3 to the *Runx2* promoter, and this function of CBX4 is neither dependent on its SUMO E3 ligase, its chromodomain, nor the PRC1 complex^12^. It is well known that Runx2 is an essential transcription factor for bone development and osteoblast differentiation and that both metastasis and resistance to chemotherapy are related to the dysregulation of Runx2 in osteosarcoma^13,14^. Here, we report that CBX4 promotes metastasis in osteosarcoma via transcriptionally upregulating *Runx2*, and that down-regulation of CBX4 by both casein kinase 1α (CK1α) and the carboxyl terminus of HSC70-interacting protein (CHIP) may provide strategies to target osteosarcoma patients with metastasis.

## Results

### CBX4 functions by transcriptionally upregulating Runx2

**Fig. 1:**
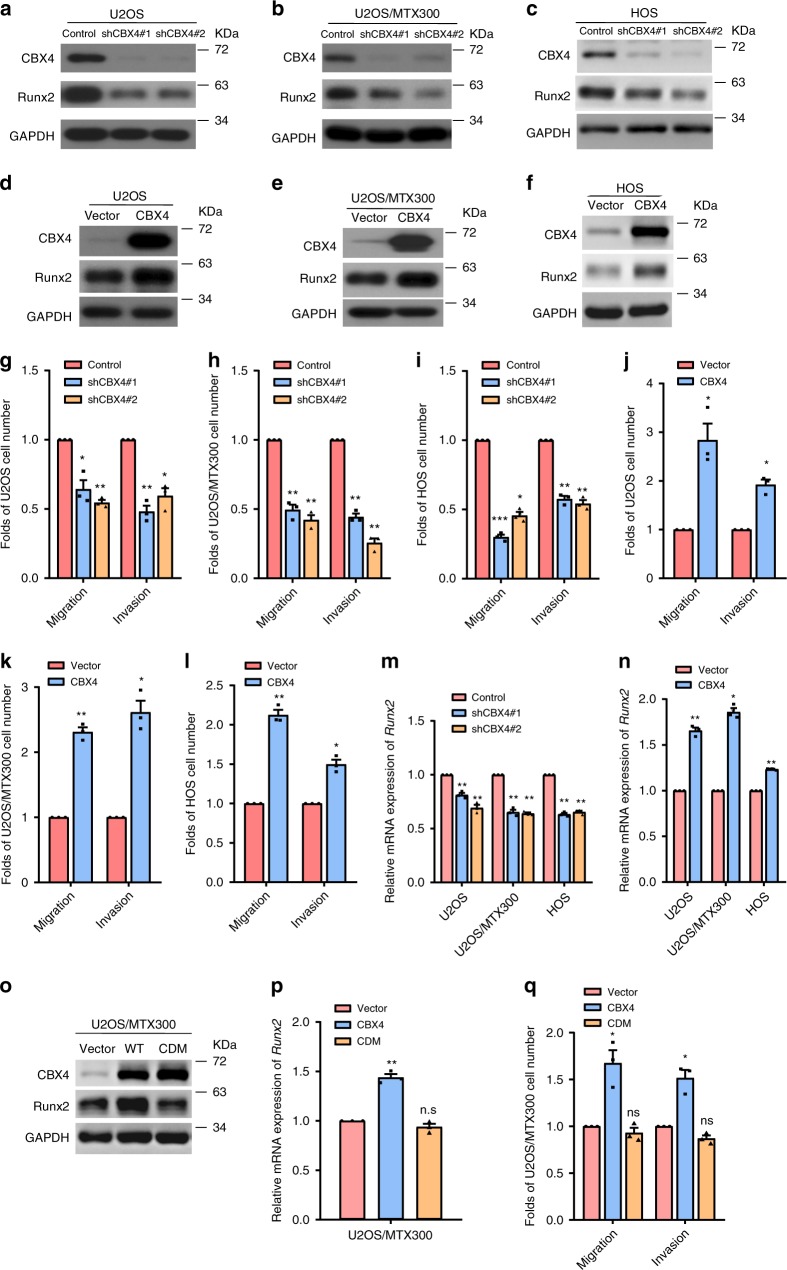
CBX4 promotes cell migration and invasion by increasing Runx2 in osteosarcoma cells. **a**–**f**, **o** The indicated proteins were analyzed by Western blotting in the indicated stable cells of three independent experiments. **g**–**l**, **q** Migration and invasion abilities were determined using the indicated stable cells as described in Methods section. **m**, **n**, **p** The relative mRNA levels of *Runx2* were normalized to the *GAPDH* level in the indicated stable cells as determined by qRT-PCR. The bars indicate the SD. The results are expressed as the mean ± SD of three independent experiments. *p < 0.05, **p < 0.01, ***p < 0.001 using the two-sided Student’s *t*-test. n.s, no significance. Source data are provided as a Source Data file.

To identify the key molecules in the progression of osteosarcoma, RNA-seq was recently performed using 16 osteosarcoma tissues and 4 normal tissues (Supplementary Table 1)^15^. We found that CBX4 and CBX8, but not CBX2 or CBX6, was overexpressed in all 16 osteosarcoma tissues compared with the 4 normal tissues (Supplementary Fig. 1A). CBX4 was also overexpressed in osteosarcoma tissues compared with normal tissues in the recently published osteosarcoma RNA-seq data (Supplementary Fig. 1B)^16^. In addition, CBX4 was overexpressed in the tested osteosarcoma cell lines compared to the osteoblast cell line hFOB1.19 (Supplementary Fig. 1C). These results argue that CBX4 may play critical roles in the progression of osteosarcoma. Indeed, knockdown of CBX4 by two pairs of small hairpin RNAs (shRNAs) reduced cell migration and invasion in U2OS, U2OS/MTX300 and HOS cells (Fig. 1a–c, g–i). Consistently, cell migration and invasion were enhanced in these cells stably expressing CBX4 (Fig. 1d–f, j–l). However, CBX4 had no effect on cell viability (Supplementary Fig. 1D–G).

We have recently reported that CBX4 is a tumor suppressor by impairing *Runx2* transcription via recruiting HDAC3 to the *Runx2* promoter in CRC, while *Runx2* plays a crucial role in promoting osteosarcoma metastasis^12,13^, which was verified by our experiments, as shown in Supplementary Fig. 2. Therefore, we were curious whether CBX4 could also affect *Runx2* transcription in osteosarcoma. The protein and mRNA levels of Runx2 were decreased by knocking down CBX4, while overexpression of CBX4 increased the protein and mRNA levels of Runx2 in U2OS, U2OS/MTX300, and HOS cells (Fig. 1a–f, m, n). However, the chromodomain mutant of CDM-CBX4 could not increase Runx2 at both mRNA and protein levels, neither migration nor invasion (Fig. 1o–q), and depletion of Ring1b did not impact the down-regulation of Runx2 induced by CBX4 knockdown (Supplementary Fig. 3A), indicating that such effects of CBX4 is dependent on its chromodomain, but not the PCR1 complex, in osteosarcoma cells. In addition, depletion of other CBX family members, such as CBX2 and CBX6, or PRC1 core subunits, Ring1a and Ring1b, did not consistently show to affect the mRNA level of Runx2 in these osteosarcoma cell lines (Supplementary Fig. 3B–E). Collectively, these results indicate that CBX4 is playing an important role in regulating Runx2 as well as cell migration and invasion in osteosarcoma cells.

**Fig. 2:**
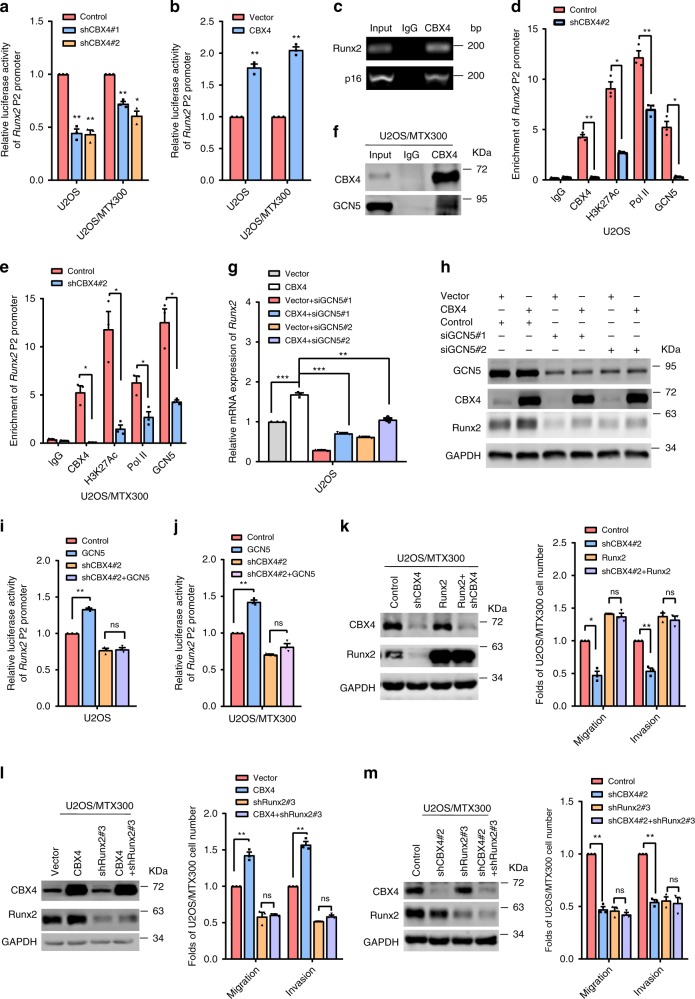
CBX4 increases Runx2 via recruiting GCN5 to the *Runx2* promoter in osteosarcoma cells. **a**, **b** The indicated stable cells transfected with the *Runx2*-Luc reporter for 48 h were subjected to the luciferase activity assay as described in Methods section. **c** The ChIP assay was performed in U2OS cells using anti-CBX4 antibody or IgG antibody, as indicated, of three independent experiments. The *p16* promoter was used as the positive control. **d**, **e** ChIP-qPCR analysis of the occupancies of CBX4, GCN5, H3K27Ac, and Pol II on the *Runx2* promoter in the indicated stable cells. **f** U2OS/MTX300 cells were subjected to immunoprecipitation (IP) using anti-CBX4 antibody or anti-IgG antibody followed by Western blotting as indicated of three independent experiments. **g**, **h** U2OS cells expressing pSIN-Vector or pSIN-CBX4 were transiently transfected with siGCN5 as indicated and then analyzed by qRT-PCR (**g**) and Western blotting (**h**). **i**, **j** The indicated stable cells co-transfected with the *Runx2*-Luc reporter and HA-GCN5 for 48 h were subjected to the luciferase activity assay as described in Methods section. **k**–**m** The indicated U2OS/MTX300 cells were subjected to Western blotting, cell migration and invasion assays. The bars indicate the SD. The results are expressed as the mean ± SD of three independent experiments. *p < 0.05, **p < 0.01, ***p < 0.001 using the two-sided Student’s *t*-test. n.s no significance. Source data are provided as a Source Data file.

Next, the knocking down or overexpression of CBX4 could inhibit and increase the *Runx2* promoter activity, respectively (Fig. 2a, b). As shown in Fig. 2c and Supplementary Fig. 4A, using the *p16 (INK4a/ARF)* promoter as the positive control, CBX4 was connected with the *Runx2* P2 promoter based on the chromatin immunoprecipitation (ChIP) assay. Using RNA polymerase II (Pol II) as the positive control, the association of H3K27Ac to the *Runx2* promoter was impaired by knocking down CBX4 in both U2OS and U2OS/MTX300 cells (Fig. 2d, e). These results were contrast to that in HCT116 cells, in which the binding of H3K27Ac in the *Runx2* promoter was increased by knocking down CBX4^12^, indicating that the opposite effects of CBX4 on Runx2 between osteosarcoma cells and CRC cells may be due to the different status of H3K27Ac in the *Runx2* promoter. In fact, overexpression of HDAC1, HDAC2, and HDAC3, which were easily detected in these osteosarcoma cell lines (Supplementary Fig. 4B), had no effect on the Ru*nx2* promoter activity in U2OS cells (Supplementary Fig. 4C), whereas the *Runx2* promoter activity was decreased by overexpression of HDAC3 in HCT116 cells^12^. These results promoted us to identify histone acetyltransferases (HATs) that could be recruited by CBX4 to the *Runx2* promoter to increase H3K27Ac and subsequently to upregulate *Runx2* expression in osteosarcoma cells, as HATs are mainly responsible for the acetylation of histone H3K27. Co-IP showed that CBX4 bound to P300, CBP, or GCN5, but neither TIP60 nor PCAF, at their exogenous and endogenous levels (Fig. 2f, Supplementary Fig. 4D–F). Moreover, GCN5 promoted, but both P300 and CBP inhibited, the *Runx2* promoter activity in U2OS cells (Supplementary Fig. 4G), indicating that CBX4 may increase Runx2 expression by recruiting GCN5 to the *Runx2* promoter. Indeed, silencing GCN5 abolished the increase of Runx2 at both mRNA and protein levels induced by overexpression of CBX4 in U2OS cells (Fig. 2g, h). Additionally, knockdown of CBX4 not only abrogated the enhancement of *Runx2* promoter activity by GCN5 (Fig. 2i, j), but also reduced the binding of GCN5 to the *Runx2* promoter (Fig. 2d, e). Furthermore, overexpression of Runx2 reversed the suppression of cell migration and invasion induced by knocking down CBX4 (Fig. 2k), while depletion of endogenous Runx2 abolished the enhancement of both migration and invasion induced by CBX4 overexpression (Fig. 2l), indicating that the effects of CBX4 on osteosarcoma cell migration and invasion depended on Runx2. Moreover, additional depletion of Runx2 could not further decrease migration and invasion in the cells knockdown of CBX4 (Fig. 2m). Collectively, these results demonstrate that CBX4 promotes cell migration and invasion by enhancing *Runx2* expression via recruiting GCN5 to the *Runx2* promoter in osteosarcoma cells. Consistently, by the GO analysis using the RNA-seq data from U2OS/MTX300 cells overexpressing or knockdown CBX4, the genes highly related to signaling receptor activity, cell migration, and angiogenesis, but not to stem cell, were significantly altered (Supplementary Fig. 5A, B). Meanwhile, other reported CBX4 substrates, such as GATA6, HOXA2, and FN1, were downregulated by CBX4-overexpression (Supplementary Fig. 5C), while GATA4 was downregulated by CBX4-knockdown (Supplementary Fig. 5D).

### CHIP is the E3 ligase responsible for the ubiquitination and degradation of CBX4

**Fig. 3:**
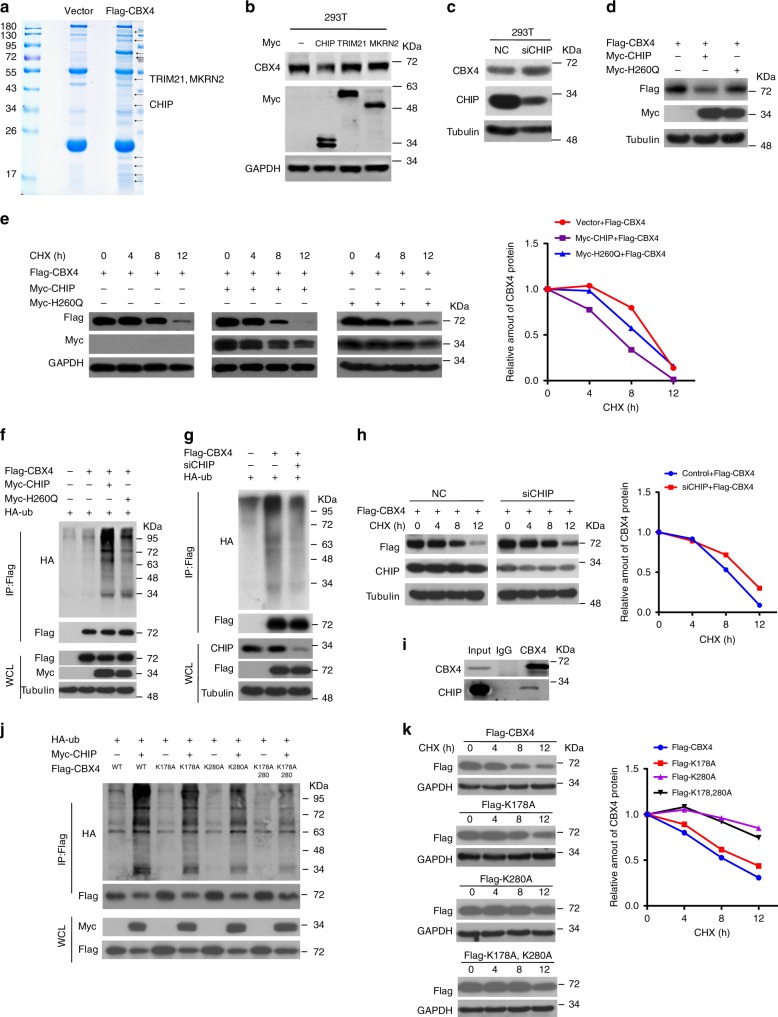
CHIP is the E3 ligase for the ubiquitination and degradation of CBX4. **a** Lysates from HEK293T cells were IP with anti-Flag agarose and subjected to SDS-PAGE and Coomassie staining. The indicated bands were sequenced by mass spectrometry (MS) analysis. **b**–**d** HEK293T cells transfected with the indicated plasmids or siRNAs for 48 h were analyzed Western blotting. These resluts are repeated of three independent experiments. **e**, **h**, **k** HEK293T cells co-transfected with the indicated plasmids for 36 h were incubated with 20 μg/ml cycloheximide (CHX) for the indicated periods and then analyzed by Western blotting. Quantitation of Flag-CBX4 protein levels was based on the Western blotting results. *n* = 1. **f**, **g**, **j** HEK293T cells were co-transfected with the indicated plasmids for 48 h and then subjected to IP using anti-FLAG antibody followed by Western blotting. **i** U2OS/MTX300 cells were subjected to IP using anti-CBX4 antibody or anti-IgG antibody followed by Western blotting as indicated. These resluts are repeated of three independent experiments. Source data are provided as a Source Data file.

Our previous data identified that the ten amino acids in the C-terminal of CBX4 may be crucial for its protein stability^12^, and CBX4 promotes cell migration and invasion in osteosarcoma cells as shown above. We sought to identify the E3 ligase responsible for degrading CBX4 by IP-mass spectrometry (MS) analysis using HEK293T cells transiently expressing Flag-tagged CBX4. There were three well-known E3 ligases, TRIM21, MKRN2 and CHIP, among the CBX4-interacting proteins (Fig. 3a, Supplementary Table 2). However, the CBX4 protein level was only reduced by CHIP, but not TRIM21 or MKRN2, at exogenous and endogenous levels (Fig. 3b, and Supplementary Fig. 6A, B), and knockdown of CHIP by siRNA or shRNA increased endogenous CBX4 protein level in cells (Fig. 3c, and Supplementary Fig. 6C). Furthermore, wild-type CHIP, but not its H260Q mutant lacking E3-ligase activity, decreased the protein level, enhanced the ubiquitination and shortened the half-life of CBX4 (Fig. 3d–f), whereas the knockdown of CHIP by siRNA decreased the ubiquitination and prolonged the half-life of CBX4 (Fig. 3g, h). The downregulation of CBX4 by CHIP was rescued by MG132 and bafilomycin (Baf), the proteasome and lysosome inhibitor, respectively (Supplementary Fig. 6D). Consistently, the interaction between CHIP and CBX4 was already detected at their exogenous and endogenous levels (Fig. 3i, and Supplementary Fig. 6E). These results reveal that CHIP is the E3 ligase responsible for the ubiquitination of CBX4 and degradation by the proteasome/lysosome.

To further identify the ubiquitination site(s) of CBX4 induced by CHIP, a set of fragments of CBX4 were produced. The ubiquitination of two separate regions of CBX4, residues 1-200aa and 251-288aa, were enhanced by CHIP (Supplementary Fig. 7A–C). Intriguingly, two lysine residues that were separated from these two regions, K178 and K280, might be ubiquitinated, as predicted by a data base analysis (Supplementary Table 3). Therefore, three CBX4 mutants, K178A, K280A, and K178/280A, were generated by replacing the lysines (K) with alanines (A). As shown in Fig. 3j, the ubiquitination of CBX4 induced by CHIP was almost abolished in the K178/280A mutant, while it was partially diminished in the mutant of either K178A or K280A. In support of these findings, these three mutants of CBX4 were more resistant to degradation by CHIP compared with wild-type CBX4, as indicated by their protein levels and half-lives (Fig. 3k, and Supplementary Fig. 7D). These results demonstrate that K178 and K280 of CBX4 are key sites for ubiquitination by CHIP.

### CK1α phosphorylates CBX4 at T437 to promote its turnover by CHIP

**Fig. 4:**
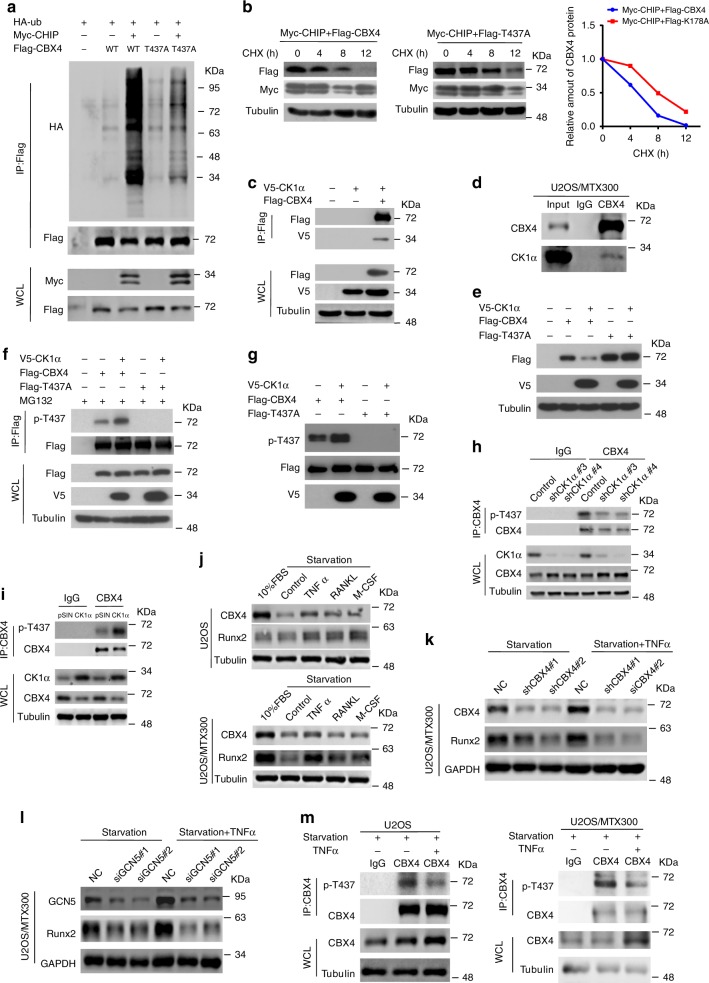
CK1α promotes the turnover of CBX4 by CHIP. **a**, **c** HEK293T cells were cotransfected with the indicated plasmids for 48 h and then subjected to IP using anti-FLAG antibody followed by Western blotting analysis of three independent experiments. **b** HEK293T cells cotransfected with the indicated plasmids for 36 h were incubated with 20 μg/ml CHX for the indicated periods and then analyzed by Western blotting. Quantitation of Flag-CBX4 protein levels was based on the Western blotting results. *n* = 1. **d** U2OS/MTX300 cells were subjected to IP using anti-CBX4 antibody or anti-IgG antibody followed by Western blotting as indicated. **e** HEK293T cells cotransfected with the indicated plasmids for 48 h were analyzed by Western blotting. **f** HEK293T cells cotransfected with the indicated plasmids for 40 h were incubated with 10 μM MG132 for 8 h and then subjected to IP using anti-FLAG antibody followed by Western blotting. **g** Flag-CBX4 WT or T437A mutant was purified from HEK293T cells, incubated with or without the purified V5-CK1α in vitro as described in Methods, and then analyzed by Western blotting. **h**, **i** The co-IP assay was performed using the indicated U2OS/MTX300 stable cells with anti-CBX4 antibody or anti-IgG antibody as indicated. **j**–**m** The indicated cells were starved by excluding fetal bovine serum (FBS) from the medium for 24 h, and then the cells were treated with TNFα, RANKL, and M-CSF for 24 h as indicated and subjected to Western blotting (**j**–**l**) or the co-IP assay using anti-CBX4 antibody or anti-IgG antibody as indicated (**m**). These resluts are repeated of three independent experiments. Source data are provided as a Source Data file.

In general, CHIP-mediated protein degradation is regulated by the phosphorylation of either itself or its substrates^17,18^. In fact, CBX4 may be phosphorylated at two sites, T184 and T437, as shown by mass spectrometry (Supplementary Table 4). The T437 mutant, but not the T184 mutant, affected CHIP-mediated CBX4 ubiquitination and degradation (Fig. 4a, b, and Supplementary Fig. 8A), strongly suggesting that T437 might be the dominant phosphorylation site required for the ubiquitin-mediated degradation of CBX4. However, the T437 mutants slightly affected their interactions with CHIP (Supplementary Fig. 8B).

Using the Protein Stability Regulators Screening Assay (Pro-SRSA) we recently generated^19^, we found that the pAd-DsRed-IRES-EGFP-CBX4 reporter worked properly using the knockdown of CHIP by siRNAs as the positive control (Supplementary Fig. 8C–E). Then, using the CRISPR-Cas9 kinase library to screen the kinase (s) that regulate CBX4 protein stability (Supplementary Fig. 8F), CK1α, but not other CK1 isoforms, was found to be the kinase that downregulated the protein stability of CBX4, which was validated by the observation that multiple sgRNAs targeting CK1α could increase the protein level of CBX4 (Supplementary Fig. 8G). This is consistent with the prediction that T437 of CBX4 is a consensus motif (S/TxxS/T), where x is any amino acid, for casein kinase 1 (CK1) (Supplementary Table 4). Indeed, the interaction between CBX4 and CK1α was detected at both exogenous and endogenous levels in cells (Fig. 4c, d). Furthermore, the protein level of wild-type CBX4 (WT-CBX4), but not its T437A mutant, was reduced by CK1α (Fig. 4e). Using an anti-p-T437 antibody (Supplementary Table 5) we generated that specifically recognizes CBX4 when it is phosphorylated at T437, the phosphorylation of CBX4 at T437, but not its T437A mutant, was increased by co-transfection with CK1α in cells (Fig. 4f); after incubation with the purified V5-CK1α in vitro, the phosphorylation of CBX4 at T437, but not its T437A mutant, was also increased (Fig. 4g). Moreover, the phosphorylation of endogenous CBX4 at T437 was reduced and enhanced by knockdown and overexpression of CK1α in U2OS/MTX300 cells, respectively, indicating that the phosphorylation of endogenous CBX4 at T437 was also dependent on CK1α (Fig. 4h, i).

Next, we sought to test whether the phosphorylation of CBX4 at T437 by CK1α is relevant to the progression of osteosarcoma. Both U2OS and U2OS/MTX300 cells were treated with several cytokines that are closely related to osteosarcoma progression, such as TNFα, RANKL, and M-CSF^20–22^. Interestingly, TNFα, but not RANKL or M-CSF, could increase protein levels of both CBX4 and Runx2 in these cells (Fig. 4j). The increase of Runx2 protein level by TNFα was dependent on CBX4 or GCN5 (Fig. 4k, l), and was probably due to the reduction of phosphorylation of CBX4 at T437 in these cells (Fig. 4m). Collectively, these results determine that phosphorylation of CBX4 at T437 by CK1α promotes its turnover by CHIP and is relevant to the progression of osteosarcoma.

### CK1α acts via inhibiting CBX4, and they are reversely correlated in tissues

**Fig. 5:**
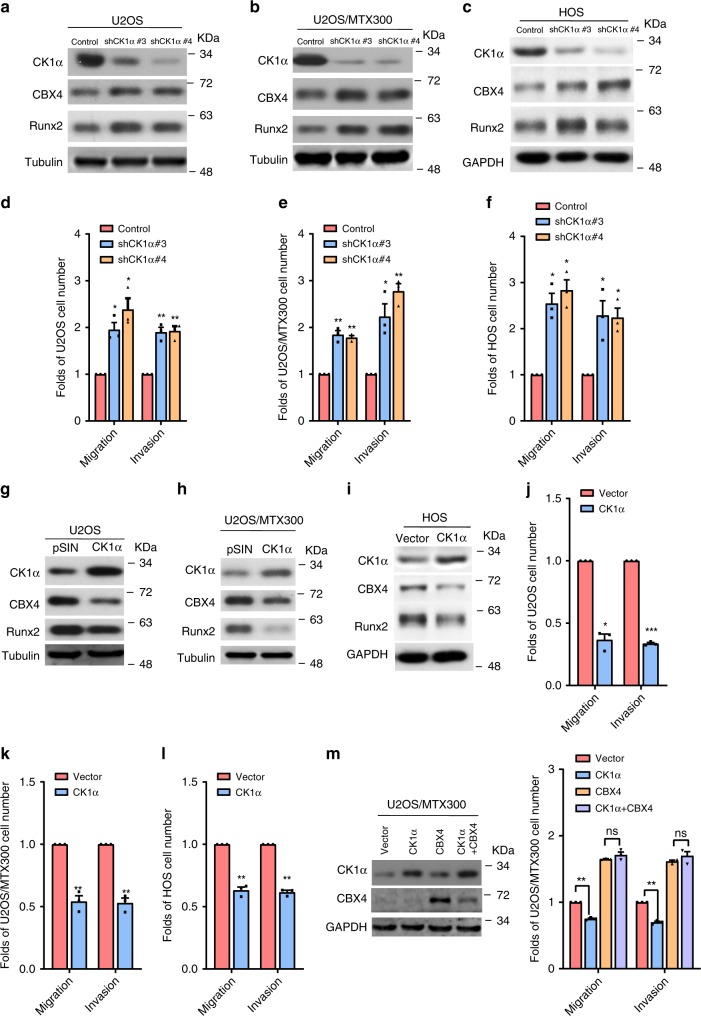
Inhibition of cell migration and invasion by CK1α primarily depends on the decrease of CBX4 in osteosarcoma cells. **a**–**c**, **g**–**i** The indicated proteins were analyzed by Western blotting in the indicated stable cells. **d**–**f**, **j**–**l** The migration and invasion abilities were determined in the indicated stable cells as described in Methods section. **m** The indicated U2OS/MTX300 cells were subjected to Western blotting, cell migration and invasion assays. The bars indicate the SD. The results are expressed as the mean ± SD of three independent experiments. *p < 0.05, **p < 0.01, ***p < 0.001 using the two-sided Student’s *t*-test. n.s no significance. Source data are provided as a Source Data file.

Then, we investigated the effects of CK1α on osteosarcoma cell migration and invasion. As shown in Fig. 5a–f, both CBX4 and Runx2 protein levels, as well as cell migration and invasion were increased by knockdown of CK1α using shRNAs in U2OS, U2OS/MTX300, and HOS cells. In contrast, stable exogenous expression of CK1α in these cells decreased the protein levels of CBX4 and Runx2, as well as cell migration and invasion (Fig. 5g–l). More importantly, overexpression of CBX4 abolished the inhibition of cell migration and invasion induced by overexpression of CK1α (Fig. 5m). These results demonstrate that overexpression of CK1α suppresses cell migration and invasion by decreasing the protein level of CBX4.

**Fig. 6:**
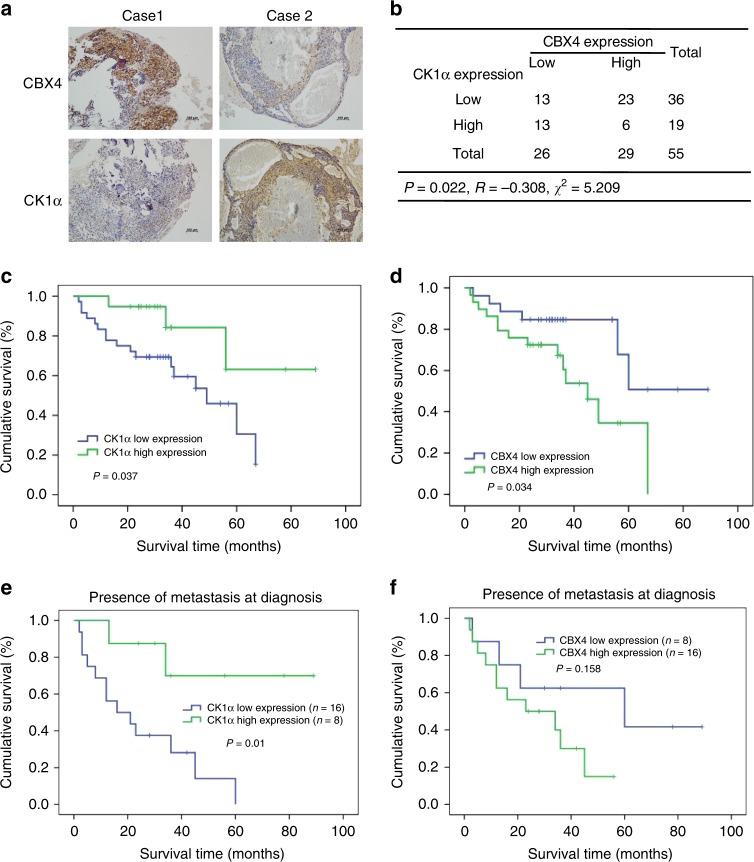
Reverse correlation between CK1α and CBX4 in osteosarcoma tissues. **a** Representative immunohistochemical staining images of both CBX4 and CK1α for 55 paraffin-embedded osteosarcoma tissues. Scale bar: 100 μm. **b** A positive correlation was observed between CBX4 and CK1α protein levels in the osteosarcoma tissues used in **a** (*p* = 0.022, *χ*^2^ tests. *R*: Spearman correlation coefficient). *p < 0.05 using the two-sided Pearson chi-squared tests. **c**–**f** Overall survival curves were generated based on the protein levels of CK1α (**c**, **e**) or CBX4 (**d**, **f**) in the osteosarcoma tissues used in **a**. *p < 0.05 using Kaplan–Meier plots and compared with the log-rank test.

To evaluate the clinical significance of the regulation of CBX4 by CK1α, the immunohistochemical (IHC) staining using both anti-CBX4 and anti-CK1α antibodies, which are highly specific^12^ (Supplementary Fig. 9), were performed in 55 osteosarcoma tissues (Supplementary Data 1). 29 of 55 tissues had high levels of CBX4, whereas 19 of 55 tissues were shown to have high levels of CK1α, and the levels of these two proteins were reversely correlated in this cohort (Fig. 6a, b). Moreover, higher levels of CK1α and CBX4 were associated with better and poorer overall survival in osteosarcoma patients, respectively (Fig. 6c, d). The primary determinant of survival in osteosarcoma is the presence of metastatic disease, therefore, we have separated the survival curves based on presence or absence of metastasis at diagnosis. As shown in the Supplementary Fig. 9B, C, the levels of CK1α and CBX4 were not associated with overall survival in patients without metastasis at diagnosis. Among 24 patients with metastasis at diagnosis, the high level of CK1α, but not of CBX4, was associated with the better overall survival (*p* = 0.01 and *p* = 0.158, respectively) (Fig. 6e), although the patients with low level of CBX4 showed the tendency to have the better survival curve compared to those with high level of CBX4, probably due to the small sample size with metastasis (24 cases) (Fig. 6f). These results indicate that CK1α was a valuable marker to predict clinical outcomes in osteosarcoma patients with metastasis.

### Pyrvinium inhibits CBX4 via CK1α activation to suppress lung metastasis of osteosarcoma

**Fig. 7:**
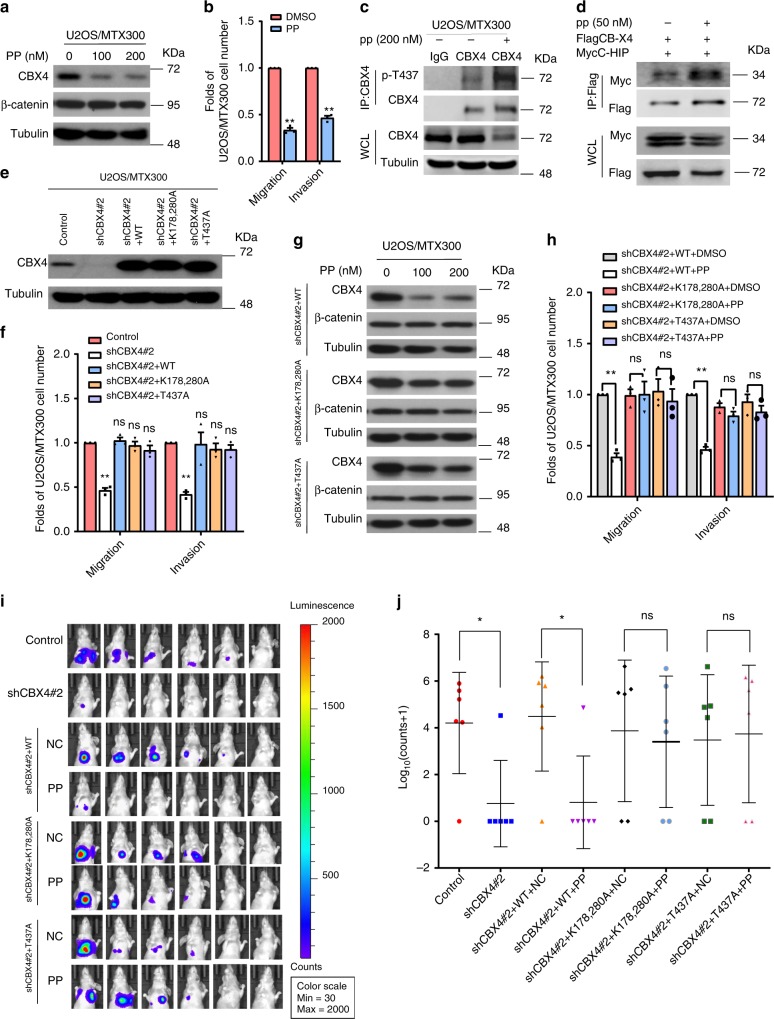
Pyrvinium suppresses lung metastasis of osteosarcoma by inhibiting CBX4 via CK1α activation. **a**, **b** The indicated cells were incubated with or without PP for 48 h and then subjected to Western blotting (**a**) and cell migration and invasion assays (**b**). **c** The indicated cells were incubated with or without PP for 48 h and then subjected to IP using anti-CBX4 antibody or anti-IgG antibody followed by Western blotting. **d** HEK293T cells transfected with the indicated plasmids 24 h were incubated with PP for 24 h and then subjected to IP using Flag-agarose followed by Western blotting. These resluts are repeated of three independent experiments. **e**, **f** The indicated U2OS/MTX300 stable cells were subjected to Western blotting (**e**) and cell migration and invasion assays (**f**). **g**, **h** The indicated U2OS/MTX300 stable cells were incubated with or without PP for 48 h and then subjected to Western blotting (**g**) and cell migration and invasion assays (**h**). *n* = 3. Data are presented as the mean ± SD of three independent experiments. *p < 0.05, **p < 0.01 using the two-sided Student’s *t*-test. n.s no significance. **i**, **j** The indicated U2OS/MTX300-luc stable cells were used in the orthotopic osteosarcoma metastasis model with or without pp (0.5 mg/kg, three times a week) as described in Methods section; *n* = 6. Data are presented as mean values ± SD. *p < 0.05 using the two-sided Student’s *t*-test. n.s: no significance. Source data are provided as a Source Data file.

Given that ubiquitin-mediated proteolysis of CBX4 is dependent on its T437 phosphorylation by CK1α, and that CBX4 promotes osteosarcoma cell migration and invasion, drugs that potentiate CK1α kinase activity should be beneficial to osteosarcoma patients with metastasis. Pyrvinium pamoate (PP) is a FDA-approved drug that selectively activates the CK1α isoform and has antitumor effects in different cancer models^23^. Therefore, we were very curious to determine whether PP could have any effect on osteosarcoma metastasis. PP inhibited endogenous protein levels of CBX4, but not β-catenin, which is also a substrate of CK1α, in U2OS/MTX300 cells (Fig. 7a). In the meanwhile, cell migration and invasion were impaired, whereas the phosphorylation of endogenous CBX4 at T437 was increased in cells treated with PP (Fig. 7b, c). Consistently, the interaction of CBX4 with CHIP was also increased by the PP treatment in cells (Fig. 7d).

Finally, the effects of PP on the orthotopic osteosarcoma metastasis model were explored in vivo using U2OS/MTX300-luci cells. As shown in Fig. 7e–j, knockdown of endogenous CBX4 by shRNA decreased cell migration, invasion and metastasis, which were completely rescued by the reintroduction of wild-type CBX4 (WT), as well as its mutant of either K178/280A or T437A, into these cells. As shown in Fig. 7g, more significant inhibition of CBX4 protein level on WT-CBX4 was observed under the PP treatment compared to those on its K178/280A and T437A mutants. Consistently, cell migration, invasion, and metastases were impaired by PP treatment in U2OS/MTX300 cells stably expressing shRNA and WT-CBX4, but not its K178/280A or T437A mutants (Fig. 7h–j). These results demonstrate that PP diminishes the metastasis of osteosarcoma by inducing the degradation of CBX4 protein, indicating that PP may be valuable for use in clinical trials for osteosarcoma patients with lung metastases.

## Discussion

**Fig. 8:**
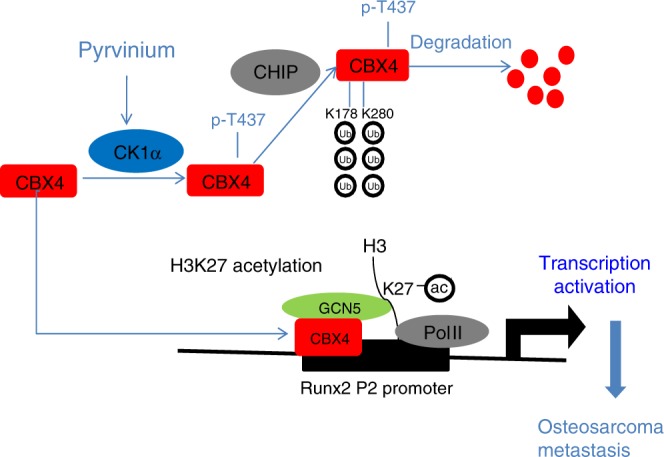
A proposed model for both function and regulation of CBX4 in osteosarcoma. CBX4 is overexpressed in osteosarcoma and recruits GCN5 to sustain H3K27Ac in the *Runx2* promoter to transcriptionally up-regulate*Runx2* and then to promote lung metastasis; the phosphorylation of CBX4 at T437 by CK1α, which is a tumor suppressor in osteosarcoma, facilitates its ubiquitination and degradation by CHIP; PP as a selective activator of CK1α promotes the degradation of CBX4, which may benefit osteosarcoma patients with high expression levels of CBX4.

In this study, as illustrated in Fig. 8, we provide evidence that overexpression of CBX4 recruits GCN5 to sustain H3K27Ac in the *Runx2* promoter to transcriptionally upregulate *Runx2* and then to promote lung metastasis in osteosarcoma. The phosphorylation of CBX4 at T437 by CK1α facilitates its ubiquitination and degradation by CHIP, and PP as a selective activator of CK1α may benefit osteosarcoma patients with high expression levels of CBX4.

CBX4 is one of the five human homologs of *Drosophila* Pc protein^24^. Traditionally, the PRC1 complex binds to H3K27me3 via its CBX proteins, and the complex then mediates transcriptional repression^25^. Therefore, the functions of CBX4 are mostly dependent on the chromodomain, which has a high binding affinity to H3K27me3. However, our group has recently shown that CBX4 impairs *Runx2* expression in CRC via recruiting HDAC3 to inhibit H3K27Ac at the *Runx2* promoter, consequently impairing CRC metastasis^12^. The recruitment of HDAC3, as well as the suppression of cancer metastasis by CBX4, are neither dependent on its chromodomain nor its PRC1 complex. Intriguingly, we showed herein that CBX4 could promote *Runx2* transcription in osteosarcoma by recruiting GCN5 to sustain H3K27Ac at the *Runx2* promoter, consequently facilitating osteosarcoma metastasis. These findings are quite interesting, as CBX4 targeting the *Runx2* promoter has two opposite effects via recruiting HDAC3 and GCN5 to alter the status of H3K27Ac to impair and enhance Runx2, which in turn inhibits and promotes CRC and osteosarcoma metastases, respectively. These findings argue that the roles of CBX4 in different types of cancer largely rely on its binding partner, even if it has the same target in different cancers. In fact, CBX4 could bind to both P1 and P2 promoters of Runx2 and had a better binding affinity to the P2 promoter in both U2OS and HCT116 cell lines (Supplementary Fig. 10A). As shown in Supplementary Fig. 10B, C, the interactions of CBX4 with both GCN5 and HDAC3 were not affected by its CDM-CBX4 mutant, whereas its ΔTM-CBX4 mutant, which lacks its binding to HDAC3^12^, had an higher binding affinity to GCN5 compared with its WT-CBX4, indicating that GCN5 may compete with HDAC3 to bind CBX4. Therefore, the effects of CBX4 on Runx2 are different among cancer cell types, depending on the cellular ratio of GCN5 to HDAC3, and subsequently CBX4 may promote or inhibit metastasis depending on the cancer type.

CK1 belongs to the serine/threonine kinases that are involved in several cellular growth and survival processes, including Wnt, Hedgehog and p53 signaling^26^. Seven CK1 isoforms (α, β, γ1, γ2, γ3, δ, and ε) and several splice variants of CK1α, δ, ε, and γ3 have been identified thus far^27^. CK1α, encoded by *CSNK1A1*, is a multifunctional protein that regulates multiple signaling pathways. For instance, CK1α targets p53 for degradation, which is mediated by murine double minute clone 2 (MDM2) and MDM4^28–31^; CK1α phosphorylates β-catenin at Ser45, leading to its recognition by E3 ubiquitin protein ligase (β-TrCP) and eventually proteasomal degradation^32,33^; CK1α has also been shown to have dual gating functions by first promoting and then terminating T cell receptor (TCR)-induced nuclear factor κB (NF-κB) activation^34^. The functions of CK1α in cancers depend on the cell type; it is likely an oncogene in colorectal cancer^35^, while it may be a tumor suppressor in lung cancer^36^ and melanoma^37^. Here we demonstrated that CK1α could inhibit cell migration and invasion by down-regulating CBX4, and that a low level of CK1α was associated with better overall survival in osteosarcoma, indicating that CK1α might be a tumor suppressor in osteosarcoma. This is also supported by a previous report showing that CBX4 is positively correlated to tumor growth and survival in osteosarcoma^38^.

In addition, we also showed that CK1α was able to phosphorylate CBX4 at T437, promoting ubiquitination and degradation of CBX4 by CHIP, consistent with the notion that phosphorylation affects the functions of the PRC1 complex subunits, such as CBX2, CBX7, and Bmi1, through different mechanisms. Notably, the cytokine TNFα, which is related to osteosarcoma progression, could reduce the phosphorylation of CBX4 at T437 to increase CBX4 expression in cells, suggesting that the phosphorylation of CBX4 at T437 by CK1α is relevant to the development of osteosarcoma. This may also indicate that targeting TNFα pathway may benefit osteosarcoma patients with metastasis, as TNFα may increase CBX4 to promote osteosarcoma metastasis.

PP as a selective activator of CK1α is a quinoline-derived cyanine dye and an oral FDA-approved anthelminthic drug for the treatment of *Enterobiusvermicularis* (pinworm), and it has garnered considerable attention due to its anti-tumor effect^23^. For instance, PP treatment at nanomolar concentrations can reduce the proliferation of various CRC cell lines via suppression of β-catenin/Tcf4^39^; PP inhibits the growth of HER2-positive breast cancer cells carrying PI3KCA mutations by inhibiting the phosphorylation of S6 and AKT proteins^40^; PP suppresses prostate cancer cell growth through direct targeting endogenous AR in LAPC4 and LNCaP cell lines, as well as in mouse prostate^41,42^. In addition, PP has been shown to function as a therapeutic agent for the treatment of early malignant tumors and progressive metastatic cancers through selectively targeting cancer stem cells by inhibiting the Wnt signaling pathway^43,44^. We demonstrated herein that PP could impede lung metastasis using the transplanted xenograft in the orthotopic osteosarcoma metastasis model. Our findings may be clinically meaningful, as CBX4 is overexpressed in most osteosarcoma patients (e.g., 29 out of 55) and promotes metastasis. We strongly recommend the investigation of PP or its derivatives in a clinical trial for the treatment of osteosarcoma patients with high expression levels of CBX4.

## Methods

### Cell culture

The hFOB1.19, U2OS, MG63, 143B, HOS, and HEK293T embryonic kidney cells were obtained from the American Type Culture Collection (ATCC) and cultured according to the instructions from the ATCC. The ZOS and ZOS-M, syngeneic human osteosarcoma cell lines derived from a primary tumor and metastasis, respectively, from the same patient^45^. All of the cell lines and the primary cell cultures were grown in Dulbecco’s modified Eagle medium (Invitrogen, Grand Island, NY) supplemented with 10% fetal bovine serum (Invitrogen) at 37 °C and 5% CO_2_. Starting with U2OS osteosarcoma cell line, the MTX300-resistant variant was obtained by exposing the parental line in vitro to stepwise the increased MTX concentrations. Such continuous exposure to MTX resulted in cells resistant to 300 µg/l (U2OS/MTX300). The MTX300-resistant variant was continuously cultured in the presence of 300 µg/l MTX^46,47^.

### Plasmid construction

The Flag-tagged HDAC constructs were gifts from Prof. Binhua P. Zhou (University of Kentucky). The HA-tagged *P300*, *CBP*, *TIP60*, *GCN5*, and *PCAF*, Flag tagged-*CBX4*, Myc-tagged *CHIP*, *TRIM21*, and *MKRN2* and V5-tagged *CK1α* were cloned into the PCDNA3.1 vector.

*CBX4, Runx2*, and *CK1α* were cloned into the pSIN-EF2-puro vector. The promoter regions of *Runx2* were cloned into the pGL3-basic vector. The PLKO.1-puro vector was used to clone the shRNAs targeting *CBX4* and *CK1α*. The sequences used for cloning the indicated shRNAs are shown in Supplementary Materials.

### RNA extraction and qRT-PCR

Briefly, total RNA was isolated using TRIzol reagent (Invitrogen) according to the manufacturer’s instructions. First-strand cDNA was synthesized using the Revert AidTM First Strand cDNA Synthesis Kit (MBI Fermentas). The primers used to amplify the indicated genes are shown in Supplementary Materials.

### RNAi treatment

The oligonucleotide sequences targeting *GCN5* and *CHIP* mRNA were as follows: GCN5: #1 CCAACTGTCGCGAGTACAA, #2 GAAGCTGATTGAGCGCAAA; CHIP: TGCCGCCACTATCTGTGTA.

Transfection was performed according to the manufacturer’s instructions using Lipofectamine RNAi MAX transfection reagent (Invitrogen) and 50 nM siRNA.

### The luciferase reporter assay

Briefly, the cells were plated in 12-well plates at a density of 1.4 × 10^5^ cells per well and then transfected with 0.8 μg of promoter-luciferase plasmid. To normalize the transfection efficiency, the cells were also cotransfected with 8 ng of pRL-CMV (Renilla luciferase). After transfection for 48 h, luciferase activity was measured using a Dual-Luciferase Assay kit (Promega). Three independent experiments were performed, and the calculated means and standard deviations are presented. The primers used for cloning the Runx2 P2 promoter are shown in Supplementary Materials.

### Western blotting and co-immunoprecipitation (Co-IP)

Briefly, cells were collected, lysed in RIPA buffer (150 mM NaCl, 0.5% EDTA, 50 mM Tris, 0.5% NP40) and centrifuged for 20 min at 13,500 × *g* and 4 °C. Fifty micrograms of harvested total protein were loaded and separated on an 8% sodium dodecyl sulfate–polyacrylamide gradient gel. The proteins were then transferred onto PVDF membranes and blocked with 5% nonfat milk for 2 h at room temperature. The membranes were incubated with primary antibody and horseradish peroxidase-conjugated secondary antibody, and the proteins were then detected using the ECL chemiluminescence system (Pierce, Rockford, USA).

For Co-IP, the clarified supernatants were first incubated with anti-HA-agarose (Sigma Aldrich), anti-Myc-agarose, or anti-FLAG-agarose (Sigma Aldrich) gels for 2 h to overnight at 4 °C, and the precipitates were washed five times with RIPA. To investigate the interaction between endogenous CBX4 and GCN5, CHIP, or CK1α, the clarified supernatants were first incubated with an anti-CBX4 antibody for 2 h at 4 °C. Protein A/G-agarose was then added for 2 h to overnight, and the precipitates were washed five times with RIPA and analyzed by Western blotting. The antibodies used in this work are shown in Supplementary Materials and the p-T437(CBX4) antibody is dilluted at 1:1000.

### Mass spectrometry (MS) analysis

Affinity purification of Flag-tagged-CBX4 was carried out. Briefly, HEK293T or U2OS/MTX300 cells were transfected with plasmids encoding Flag-tagged CBX4. The cells were lysed in NETN buffer containing 50 mmol/l β-glycerophosphate, 10 mmol/l NaF, and 1 mg/ml each of pepstatin A and aprotinin. The lysates were centrifuged at 13,500 × *g* to remove debris and then incubated with Flag-conjugated beads for 4 h at 4 °C. The beads were washed five times with NETN buffer, the bound proteins were analyzed by SDS-PAGE, and MS was performed by PTM BioLabs. The immunocomplexes were washed four times with NETN buffer and then subjected to SDS-PAGE and Western blotting.

### RNA-seq

RNAseq of 16 osteosarcoma tissues and 4 normal tissues was performed by Novogene using Illumina X TEN. The 6GB clean data per sample was collected for RNAseq. Hg38 assembly was used for the read alignment, and gene annotation was obtained using Ensembl gene annotation version 90. Sequence data have been deposited in BioProject database (ID: PRJNA539828).

RNAseq of CBX4 overexpression or knockdown was performed by Novogene using Illumina NovoSeq 6000. The 6 GB clean data per sample was collected for RNAseq, and the clean reads were aligned to the human genome GRCh38 (Hg38) using hisat2 (version 2.0.5). Sequence data have been deposited in GEO (GSE: 140763).

### Chromatin immunoprecipitation (ChIP) assay

This procedure was performed according to instructions supplied with the ChIP kit (Millipore, 17-10085 and 17-10086). Briefly, 15 cm plates were seeded with cells of each tested cell line and allowed to grow to 70–80% confluence. To fix the cells, Complete Cell Fixative Solution (1/10 the volume of the growth medium volume) was added to the existing culture medium. The fixation reaction was stopped by adding Stop Solution (1/20 the volume of the growth medium volume) to the existing culture medium. The cells were collected by centrifugation, and the nuclear pellet was resuspended in ChIP Buffer. The cell lysate was subjected to sonication and then incubated with 5 μg of antibody overnight, followed by incubation with the protein A/G agarose beads overnight at 4 °C. Bound DNA-protein complexes were eluted, and cross-links were reversed after a series of washes. The purified DNA was resuspended in TE buffer for PCR analysis. The primers for the indicated promoters are shown in Supplementary Materials.

### MTT assay

A 3-(4,5-dimethylthiazol-2-yl)-2,5-diphenyltetrazolium bromide (MTT) assay was used to measure cell viability. Briefly, U2OS or U2OS/MTX300 cells were seeded at a density of 2000 cells per well in a 96-well microplate. The cells were incubated with MTT for 4 h, and the optical density (OD) was detected at 490 nm with the microplate reader once per day for 4 days. The results are presented as the mean ± SD of three independent experiments.

### Migration and invasion assays

Transwell assays using Boyden chambers containing 24-well transwell plates (BD Inc., USA) with an 8 μm pore size were used to evaluate the migration and invasiveness of cells. All experiments were performed in duplicate and repeated three times. For the migration assay, the cell culture inserts were seeded with 0.5 × 10^5^ (U2OS cells) or 1 × 10^5^ (U2OS/MTX300 cells) in 200 μl of serum-free DMEM without an extracellular matrix coating. DMEM containing 20% FBS was added to the bottom chamber. After approximately 24 h of incubation, the cells on the lower surface of the filter were fixed, stained, and examined using a microscope. For the invasion assay, the membrane was coated with 50 μl of 1:8 diluted Matrigel (BD Biosciences, USA). After the Matrigel had solidified at 37 °C for 2 h, 0.5 × 10^5^ (U2OS cells) or 2 × 10^5^ (U2OS/MTX300 cells) in 200 μl of serum-free DMEM were added to the cell culture inserts, whereas the lower chamber was filled with DMEM containing 20% FBS. The Boyden chamber was then incubated at 37 °C in 5% CO_2_ for approximately 24 h. The cells were then stained and observed as described for the migration assays.

### Flow cytometry

To prepare the cells for flow cytometry sorting, live cells were harvested, resuspended in phosphate-buffered saline (PBS) with 10% fetal bovine serum and filtered using a 40 μm cell strainer (BD Falcon, Franklin Lakes, NJ, USA). Cell sorting was performed using a Beckman MoFlo Cell Sorting System (Beckman Coulter, Brea, CA, USA), and flow cytometry analysis was performed using a Beckman Cytomics FC500 flow cytometry system. Using the FSC/SSC gating, debris was removed by gating on the main cell population. Isotype control stained cells were used to distinguish between background staining and specific antibody staining. The EGFP/DsRed ratio is able to reflect protein stability in cells, which indicated that cells with altered EGFP/DsRed ratios may be sorted by flow cytometry.

### Animal experiments

Animal care and experiments were performed in strict accordance with the “Guide for the Care and Use of Laboratory Animals” and the “Principles for the Utilization and Care of Vertebrate Animals” and were approved by the Animal Research Committee of Sun Yat-sen University Cancer Center.

For the orthotopic osteosarcoma metastasis model, human osteosarcoma cells were transplanted orthotopically into the bones of mice as previously described^45,48^. We used U2OS/MTX300 cells stably expressing luciferase (U2OS/MTX300-luci) and U2OS/MTX300-luci-derived cells, according to the manuscript. Two weeks after the cells were injected into the mice, they were randomly separated into two groups. Pyrvinium (P0027, Sigma) was dissolved in 50/50% DMSO/saline, and each group of mice was treated with 50/50% DMSO/saline or pyrvinium (0.5 mg/kg) by intraperitoneal (IP) delivery three times per week. When the xenografts reached 1.5 cm in diameter, the lungs of mice bearing the osteosarcoma tumor xenografts that stably expressed luciferase were analyzed using an IVIS Lumina Imaging System (Xenogen). A total of six independent biological replicates was performed for each group to confirm the findings.

### Human tissue specimens

A total of 55 paraffin-embedded primary specimens were obtained from the recruited osteosarcoma patients. The patients were diagnosed according to their clinicopathological characteristics at the First Affiliated Hospital of Sun Yat-sen University from 2007 to 2014. No patients had received radiotherapy and/or chemotherapy prior to surgery. Tumors were staged according to the Union for International Cancer Control TNM staging system. Resected specimens were macroscopically examined to determine the location and size of a tumor, and specimens for histology were fixed in 10% (vol/vol) formalin and processed for paraffin embedding. Informed consent was obtained from all patients and approved by the research medical ethics committee of Sun Yat-sen University.

### Immunohistochemistry staining (IHC)

IHC staining was performed using 3 μm sections. The primary antibodies against CBX4 or CK1α were diluted 1:400 or 1:150, respectively, and then incubated at 4 °C overnight in a humidified container. After three washes with PBS, the tissue slides were treated with a nonbiotin horseradish peroxidase detection system according to manufacturer’s instructions (Dako). IHC staining was evaluated by two independent pathologists. The protein expression levels of CBX4 and CK1α were evaluated based on thirteen scores. Generally, the CBX4 and CK1α signals were detected in the nucleus and cytoplasm, respectively. To evaluate CBX4 and CK1α, a semiquantitative scoring criterion was used in which both the staining intensity and positive areas were recorded. A staining index (values 0–12), which was obtained as the product of the intensity of positive staining (weak, 1; moderate, 2; strong, 3) and the proportion of immunopositive cells of interest (0%, 0; *<*10%, 1; 10–50%, 2; 51–80%, 3; *>*80%, 4), was calculated. The immunohistochemical cut-off for high or low expression of the indicated molecule was determined based on the ROC curve analysis. The sensitivity and specificity for discriminating dead or alive was plotted as the IHC score, thus generating a ROC curve. The cut-off value was established as the point on the ROC curve where the sum of sensitivity and specificity was maximized. Cancers with scores above the obtained cut-off value were considered to have high expression of the indicated molecule and vice versa.

### Statistical and reproducibility

SPSS software (version 16.0, SPSS Inc., Chicago, IL, USA) was used for the statistical analysis. The significance of the differences was assessed using the two-tailed Student’s *t*-test or a chi-squared test, as appropriate. The relationship between CBX4 expression and CK1α expression was examined using Pearson chi-squared tests. The correlations between CBX4 expression, CK1α expression and overall survival curves were assessed using Kaplan–Meier plots and compared with the log-rank test. Differences were considered significant when *p* values were <0.05.

Data are presented as mean ± SD. A Student’s *t*-test (two-tailed) was used to compare two groups, which satisfy normal distribution with homogeneous variance. *p* values of <0.05 were considered significant, and *p < 0.05, **p < 0.01, and ***p < 0.001.

### Study approval

The animal experiments were approved by the Animal Research Committee of Sun Yat-sen University Cancer Center and were performed in accordance with established guidelines. The use of human osteosarcoma tissues was reviewed and approved by the ethical committee of Sun Yat-sen University Cancer Center, and informed consent was obtained.

### Reporting summary

Further information on research design is available in the Nature Research Reporting Summary linked to this article.

## Supplementary information

Supplementary Information

Description of Additional Supplementary Files

Supplementary Data 1

Reporting Summary

## Data Availability

The RNAseq data of 16 osteosarcoma tissues and 4 normal tissues have been deposited in the BioProject database under the accession code: PRJNA539828 https://trace.ncbi.nlm.nih.gov/Traces/sra/?study=SRP193919. The RNAseq data of CBX4 overexpression or knockdown have been deposited in the GEO database under the accession code: GSE: 140763. The source data underlying Figs. 1–5,  7 and Supplementary Figs. 1–10 is provided as Source Data file. All the other data supporting the findings of this study are available within the article and its supplementary information files and from the corresponding author upon reasonable request. A reporting summary for this article is available as a Supplementary Information file.

## Source Data

Source Data

## References

[1] Isakoff MS, Bielack SS, Meltzer P, Gorlick R (2015). Osteosarcoma: current treatment and a collaborative pathway to success. J. Clin. Oncol..

[2] Kansara M, Teng MW, Smyth MJ, Thomas DM (2014). Translational biology of osteosarcoma. Nat. Rev. Cancer.

[3] Bielack SS (2002). Prognostic factors in high-grade osteosarcoma of the extremities or trunk: an analysis of 1702 patients treated on neoadjuvant cooperative osteosarcoma study group protocols. J. Clin. Oncol..

[4] Link MP (1986). The effect of adjuvant chemotherapy on relapse-free survival in patients with osteosarcoma of the extremity. N. Engl. J. Med..

[5] Meyers PA (2011). Addition of pamidronate to chemotherapy for the treatment of osteosarcoma. Cancer.

[6] Li J (2014). Cbx4 governs HIF-1alpha to potentiate angiogenesis of hepatocellular carcinoma by its SUMO E3 ligase activity. Cancer Cell.

[7] Wotton D, Merrill JC (2007). Pc2 and SUMOylation. Biochem. Soc. Trans..

[8] Kagey MH, Melhuish TA, Wotton D (2003). The polycomb protein Pc2 is a SUMO E3. Cell.

[9] Satijn DP (1997). Interference with the expression of a novel human polycomb protein, hPc2, results in cellular transformation and apoptosis. Mol. Cell. Biol..

[10] Dahiya A, Wong S, Gonzalo S, Gavin M, Dean DC (2001). Linking the Rb and polycomb pathways. Mol. Cell.

[11] Wang B (2013). Chromobox homolog 4 is correlated with prognosis and tumor cell growth in hepatocellular carcinoma. Ann. Surg. Oncol..

[12] Wang Xin, Li Liping, Wu Yuanzhong, Zhang Ruhua, Zhang Meifang, Liao Dan, Wang Gang, Qin Ge, Xu Rui-hua, Kang Tiebang (2016). CBX4 Suppresses Metastasis via Recruitment of HDAC3 to the Runx2 Promoter in Colorectal Carcinoma. Cancer Research.

[13] Martin JW, Zielenska M, Stein GS, van Wijnen AJ, Squire JA (2011). The role of RUNX2 in osteosarcoma oncogenesis. Sarcoma.

[14] Vega OA (2017). Wnt/beta-catenin signaling activates expression of the bone-related transcription factor RUNX2 in select human osteosarcoma cell types. J. Cell. Biochem..

[15] Wang S (2019). Upregulation of PCOLCE by TWIST1 promotes metastasis in osteosarcoma. Theranostics.

[16] Scott MC (2018). Comparative transcriptome analysis quantifies immune cell transcript levels, metastatic progression, and survival in osteosarcoma. Cancer Res..

[17] Kao SH (2014). GSK3beta controls epithelial-mesenchymal transition and tumor metastasis by CHIP-mediated degradation of Slug. Oncogene.

[18] Kim C, Lee J, Ko YU, Oh YJ (2018). Cyclin-dependent kinase 5-mediated phosphorylation of CHIP promotes the tAIF-dependent death pathway in rotenone-treated cortical neurons. Neurosci. Lett..

[19] Wu Y (2016). A genome-scale CRISPR-Cas9 screening method for protein stability reveals novel regulators of Cdc25A. Cell Discov..

[20] Chen Y (2015). RANKL blockade prevents and treats aggressive osteosarcomas. Sci. Transl. Med..

[21] Mori T (2013). TNFα promotes osteosarcoma progression by maintaining tumor cells in an undifferentiated state. Oncogene.

[22] Kubota Y (2009). M-CSF inhibition selectively targets pathological angiogenesis and lymphangiogenesis. J. Exp. Med..

[23] Momtazi-Borojeni AA, Abdollahi E, Ghasemi F, Caraglia M, Sahebkar A (2018). The novel role of pyrvinium in cancer therapy. J. Cell Physiol..

[24] Vandamme J, Volkel P, Rosnoblet C, Le Faou P, Angrand PO (2011). Interaction proteomics analysis of polycomb proteins defines distinct PRC1 complexes in mammalian cells. Mol. Cell. Proteom..

[25] Gil J, O’Loghlen A (2014). PRC1 complex diversity: where is it taking us?. Trends Cell Biol..

[26] Cheong JK, Virshup DM (2011). Casein kinase 1: complexity in the family. Int. J. Biochem. Cell Biol..

[27] Schittek B, Sinnberg T (2014). Biological functions of casein kinase 1 isoforms and putative roles in tumorigenesis. Mol. Cancer.

[28] Huart AS, MacLaine NJ, Meek DW, Hupp TR (2009). CK1alpha plays a central role in mediating MDM2 control of p53 and E2F-1 protein stability. J. Biol. Chem..

[29] Wu S, Chen L, Becker A, Schonbrunn E, Chen J (2012). Casein kinase 1alpha regulates an MDMX intramolecular interaction to stimulate p53 binding. Mol. Cell. Biol..

[30] Wei Xi, Wu Shaofang, Song Tanjing, Chen Lihong, Gao Ming, Borcherds Wade, Daughdrill Gary W., Chen Jiandong (2016). Secondary interaction between MDMX and p53 core domain inhibits p53 DNA binding. Proceedings of the National Academy of Sciences.

[31] Elyada E (2011). CKIalpha ablation highlights a critical role for p53 in invasiveness control. Nature.

[32] Liu C (2002). Control of beta-catenin phosphorylation/degradation by a dual-kinase mechanism. Cell.

[33] Amit S (2002). Axin-mediated CKI phosphorylation of beta-catenin at Ser 45: a molecular switch for the Wnt pathway. Genes Dev..

[34] Bidere N (2009). Casein kinase 1alpha governs antigen-receptor-induced NF-kappaB activation and human lymphoma cell survival. Nature.

[35] Richter J (2018). CK1alpha overexpression correlates with poor survival in colorectal cancer. BMC Cancer.

[36] Cai J (2018). CK1alpha suppresses lung tumour growth by stabilizing PTEN and inducing autophagy. Nat. Cell Biol..

[37] Sinnberg T (2010). Suppression of casein kinase 1alpha in melanoma cells induces a switch in beta-catenin signaling to promote metastasis. Cancer Res..

[38] Yang J (2016). Chromobox homolog 4 is positively correlated to tumor growth, survival and activation of HIF-1alpha signaling in human osteosarcoma under normoxic condition. J. Cancer.

[39] Thorne CA (2010). Small-molecule inhibition of Wnt signaling through activation of casein kinase 1alpha. Nat. Chem. Biol..

[40] Carrella D (2016). Computational drugs repositioning identifies inhibitors of oncogenic PI3K/AKT/P70S6K-dependent pathways among FDA-approved compounds. Oncotarget.

[41] Jones JO (2009). Non-competitive androgen receptor inhibition in vitro and in vivo. Proc. Natl Acad. Sci. USA.

[42] Lim M (2014). Ligand-independent and tissue-selective androgen receptor inhibition by pyrvinium. ACS Chem. Biol..

[43] Lamb R (2015). Antibiotics that target mitochondria effectively eradicate cancer stem cells, across multiple tumor types: treating cancer like an infectious disease. Oncotarget.

[44] Zhang X (2015). Wnt blockers inhibit the proliferation of lung cancer stem cells. Drug Des. Devel. Ther..

[45] Tang QL (2012). Glycogen synthase kinase-3beta, NF-kappaB signaling, and tumorigenesis of human osteosarcoma. J. Natl Cancer Inst..

[46] Yin J-q (2007). Bufalin induces apoptosis in human osteosarcoma U-2OS and U-2OS methotrexate300-resistant cell lines. Acta Pharmacol. Sin..

[47] Serra M (1993). Establishment and characterization of multidrug-resistant human osteosarcoma cell lines. Anticancer Res..

[48] Berlin O (1993). Development of a novel spontaneous metastasis model of human osteosarcoma transplanted orthotopically into bone of athymic mice. Cancer Res..

