# Supplementary material for: Targeting the CK1α/CBX4 axis for metastasis in osteosarcoma
Source: Nat Commun. 2020 Feb 28;11:1141. doi: 10.1038/s41467-020-14870-4 (PMC7048933; doi:10.1038/s41467-020-14870-4)
Supplement: Supplementary file 1 — Supplementary Information [file 41467_2020_14870_MOESM1_ESM.pdf]

Supplementary Information

Targeting the CK1α/CBX4 Axis for Metastasis in Osteosarcoma

Wang et al.

Supplementary Figures 1 – 10

Supplementary Tables 1 - 5

Supplementary Materials

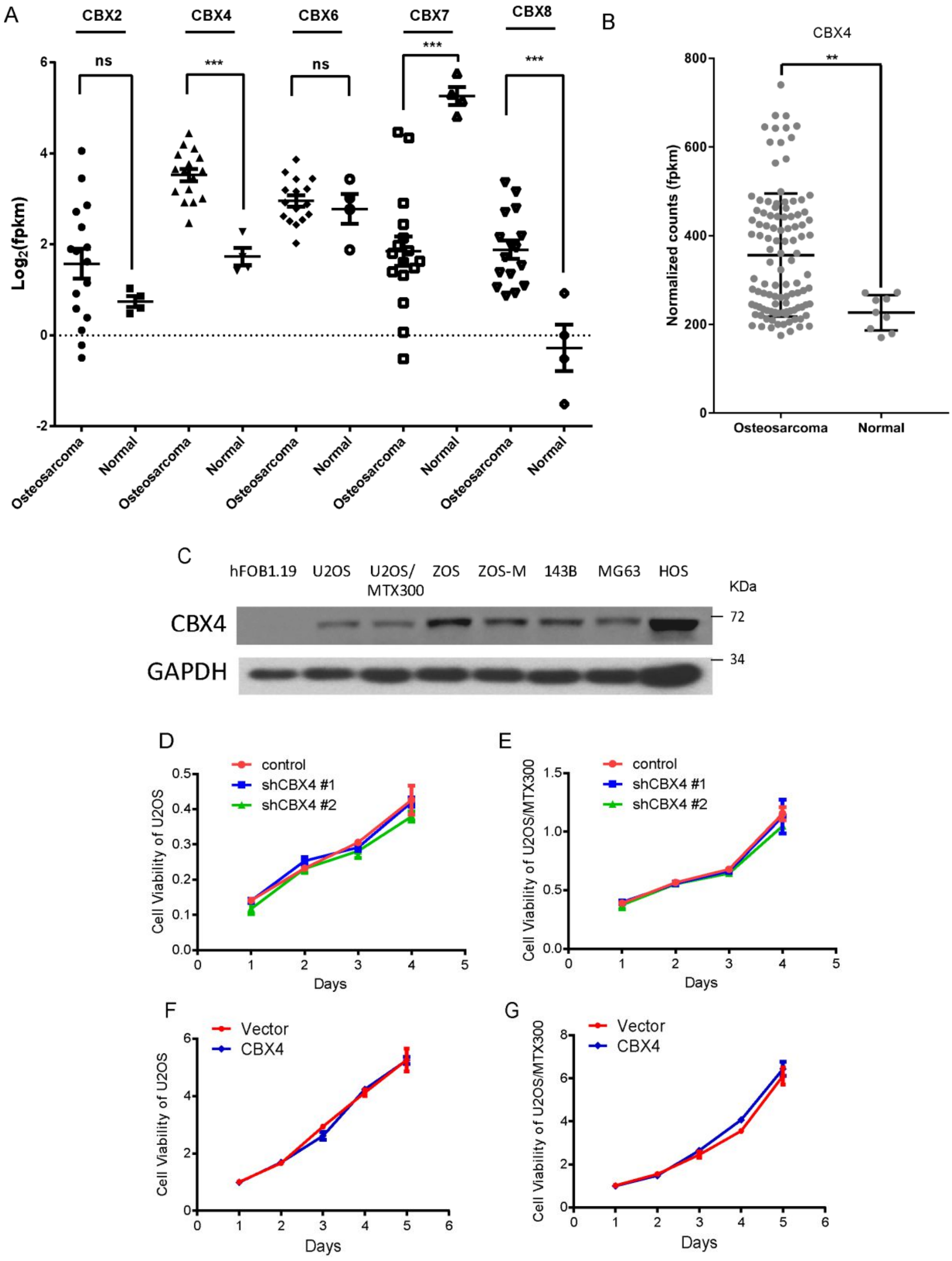
A
CBX2
CBX4
CBX6
CBX7
CBX8
ns
***
ns
***
***
Log2(fpkm)
Osteosarcoma
Normal
B
CBX4
**
Normalized counts (fpkm)
Osteosarcoma
Normal
C
hFOB1.19
U2OS
U2OS/MTX300
ZOS
ZOS-M
143B
MG63
HOS
KDa
CBX4
GAPDH
72
34
D
control
shCBX4 #1
shCBX4 #2
Cell Viability of U2OS
Days
E
control
shCBX4 #1
shCBX4 #2
Cell Viability of U2OS/MTX300
Days
F
Vector
CBX4
Cell Viability of U2OS
Days
G
Vector
CBX4
Cell Viability of U2OS/MTX300
Days

Supplementary Figure 1. Alteration of CBX4 does not affect cell viability in osteosarcoma cells. (A, B) The statistical results of CBX4 mRNA levels from our RNA-seq data for 16 osteosarcoma and 4 normal tissues **(A)** or from the published RNA-seq data for 108 osteosarcoma and 9 normal tissues **(B)**. Data are presented as mean values ± SD. **= $p < 0.01$, ***= $p < 0.001$ using the two-sided Student's *t*-test. n.s: no significance. **(C)** CBX4 protein levels were analyzed in the indicated cell lines by Western blotting of one independent experiments. **(D-G)** Cell viability was measured by the MTT assay in the indicated stable cells. The dots represent the means, and the bars indicate the SD. The results are expressed as the mean ± SD of three independent experiments. Source data are provided as a Source Data file.

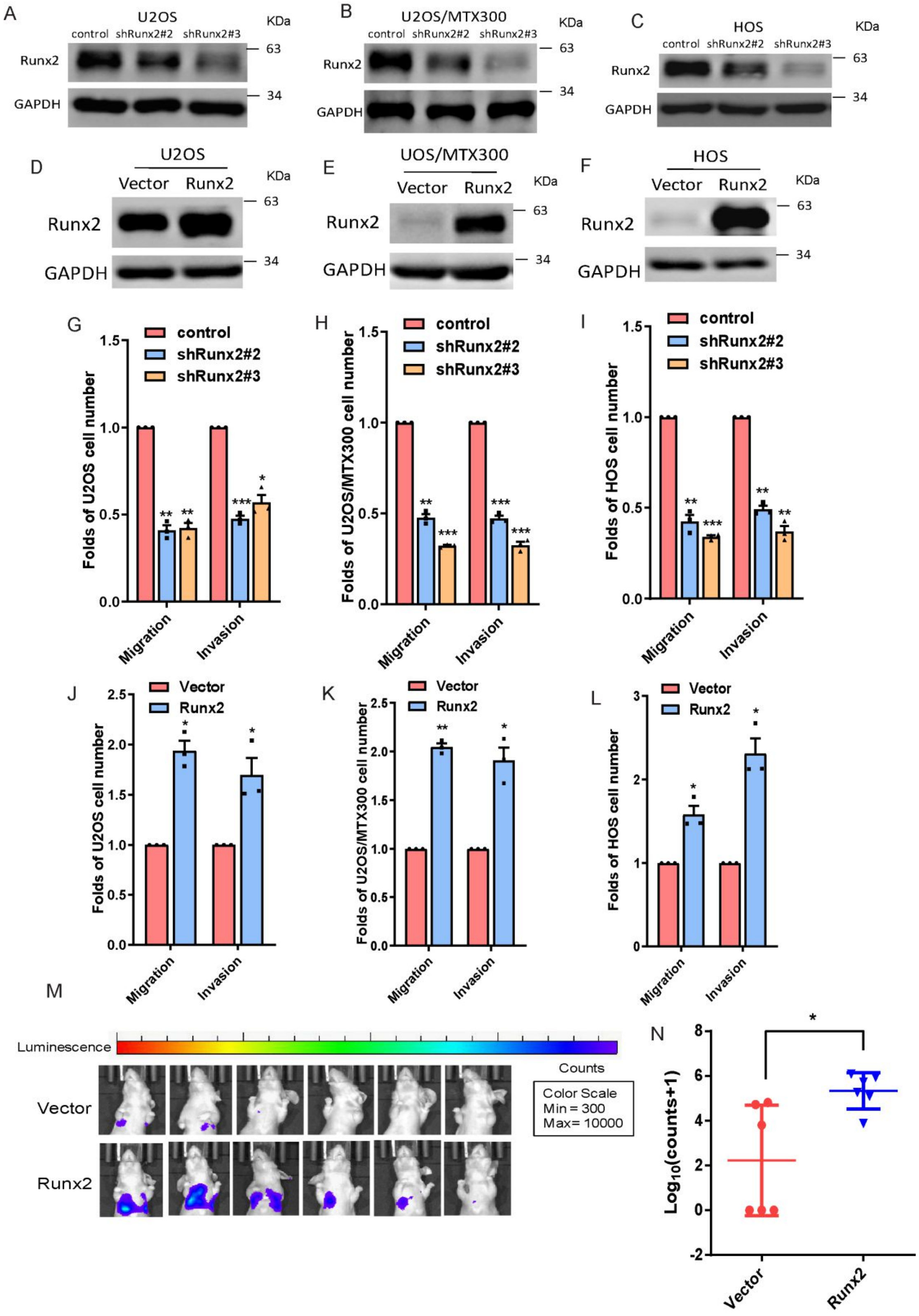

A U2OS control shRunx2#2 shRunx2#3 KDa Runx2 63 GAPDH 34
B U2OS/MTX300 control shRunx2#2 shRunx2#3 KDa Runx2 63 GAPDH 34
C HOS control shRunx2#2 shRunx2#3 KDa Runx2 63 GAPDH 34
D U2OS Vector Runx2 KDa Runx2 63 GAPDH 34
E UOS/MTX300 Vector Runx2 KDa Runx2 63 GAPDH 34
F HOS Vector Runx2 KDa Runx2 63 GAPDH 34
G control shRunx2#2 shRunx2#3 Folds of U2OS cell number Migration Invasion
H control shRunx2#2 shRunx2#3 Folds of U2OS/MTX300 cell number Migration Invasion
I control shRunx2#2 shRunx2#3 Folds of HOS cell number Migration Invasion
J Vector Runx2 Folds of U2OS cell number Migration Invasion
K Vector Runx2 Folds of U2OS/MTX300 cell number Migration Invasion
L Vector Runx2 Folds of HOS cell number Migration Invasion
M Luminescence Counts Vector Runx2
Color Scale
Min = 300
Max= 10000
N Log10(counts+1) Vector Runx2

Supplementary Figure 2. Runx2 promotes cell migration, invasion and metastasis in osteosarcoma cells. (A-F) The indicated proteins were analyzed by Western blotting in the indicated stable cells of three independent experiments. **(G-L)** The migration and invasion abilities were determined in the indicated stable cells as described in Methods. The results are expressed as the mean ± SD of three independent experiments. *= $p < 0.05$, **= $p < 0.01$, ***= $p < 0.001$ using the two-sided Student's *t*-test. **(M, N)** The indicated U2OS/MTX300-luc stable cells were used in the orthotopic osteosarcoma metastasis model as described in Methods; n= 6. Data are presented as mean values ± SD. *= $p < 0.05$ using the the two-sided Student's *t*-test. Source data are provided as a Source Data file.

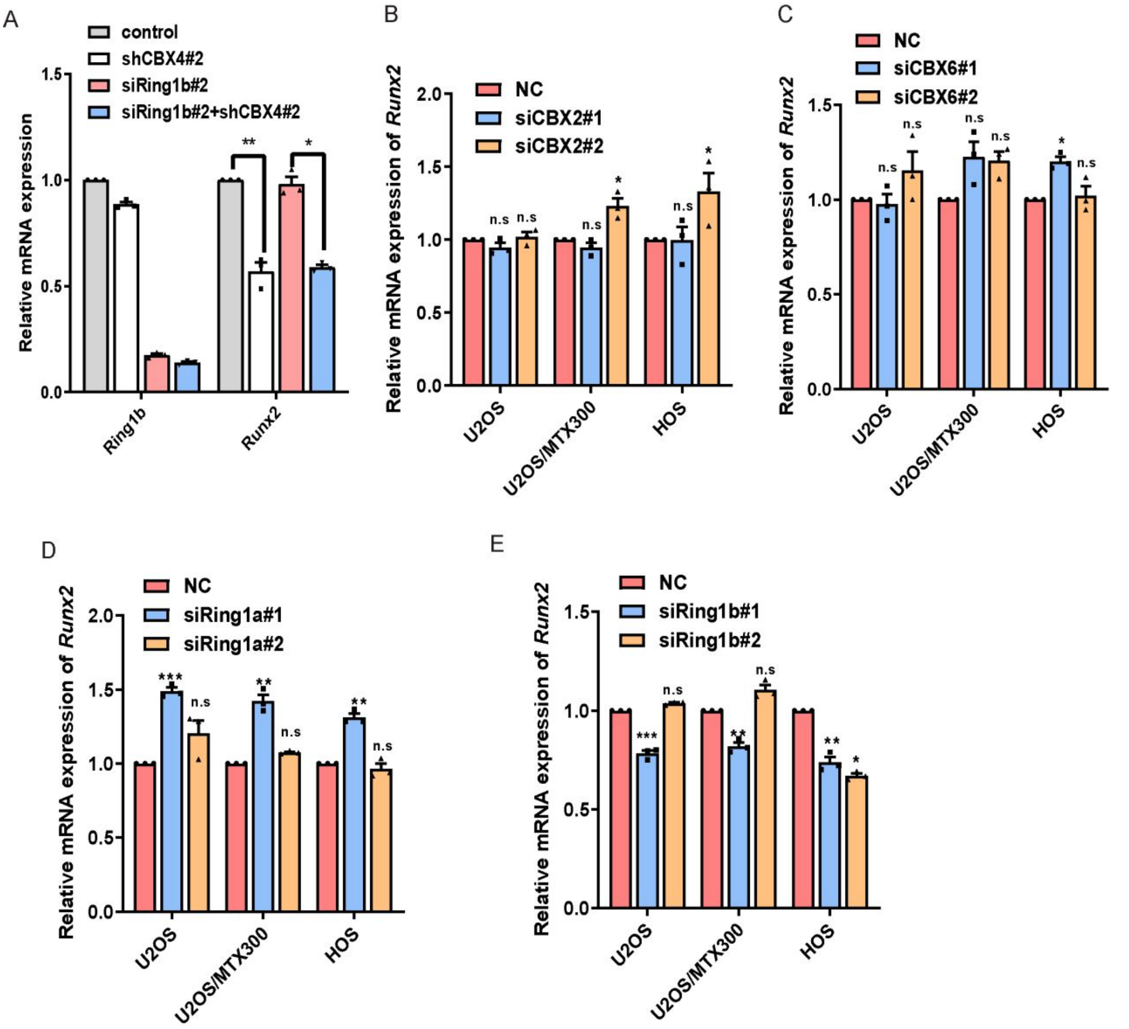

Supplementary Figure 3. Runx2 expression is independent on PRC1 complex. (A-E) The relative mRNA levels of *Runx2* or *Ring1b* were normalized to the *GAPDH* level in the indicated stable cells as determined by qRT-PCR. The bars indicate the SD. The results are expressed as the mean ± SD of three independent experiments. *= p< 0.05, **= p < 0.01, ***=p<0.001 using the two-sided Student's t-test. n.s: no significance. Source data are provided as a Source Data file.

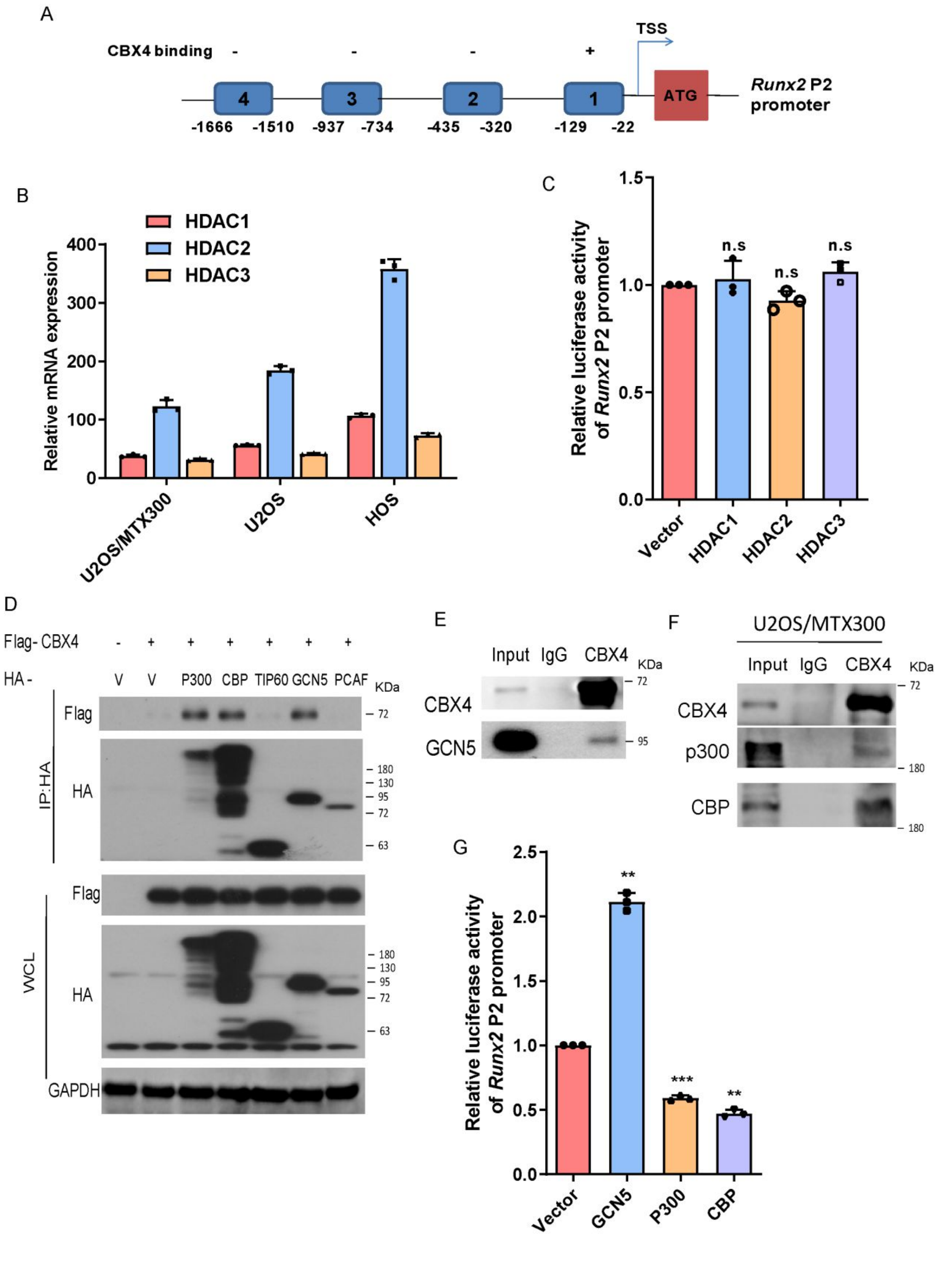
A
CBX4 binding
TSS
ATG
Runx2 P2 promoter
-1666 -1510 -937 -734 -435 -320 -129 -22
B
HDAC1
HDAC2
HDAC3
Relative mRNA expression
U2OS/MTX300
U2OS
HOS
C
Relative luciferase activity of Runx2 P2 promoter
n.s
Vector
HDAC1
HDAC2
HDAC3
D
Flag- CBX4
HA -
V
P300
CBP
TIP60
GCN5
PCAF
KDa
IP:HA
Flag
HA
WCL
GAPDH
E
Input
IgG
CBX4
GCN5
F
U2OS/MTX300
p300
CBP
G
Relative luciferase activity of Runx2 P2 promoter
Vector
GCN5
P300
CBP

Supplementary Figure 4. Runx2 expression is regulated by GCN5 but not HDAC3 in osteosarcoma cells. (A) Schematic illustration of the regions (-129 bp to -22 bp) of Runx2 promoter have a binding affinity of CBX4. **(B)** The relative mRNA levels of *HDAC1, HDAC2* or *HDAC3* were normalized to the *GAPDH* level in the indicated stable cells as determined by qRT-PCR. n=3. **(C)** U2OS cells were cotransfected with the *Runx2*-Luc reporter and Flag-HDAC1, Flag-HDAC2 or Flag-HDAC3 for 48 hours and then subjected to the luciferase activity assay as described in Methods. n=3. n.s: no significance. (**D**) HEK293T cells cotransfected FLAG-CBX4 with the indicated plasmids for 48 hours were subjected to IP using Flag-agarose followed by Western blotting. **(E and F)** HEK293T **(E)** or U2OS/MTX300 **(F)** cells were subjected to immune- precipitation (IP) using anti-CBX4 antibody or anti-IgG antibody followed by Western blotting as indicated. These resluts are repeated of three independent experiments. **(G)** U2OS cells were cotransfected with the *Runx2*-Luc reporter with the indicated plasmids for 48 hours and then subjected to the luciferase activity assay as described in Methods. n=3. The bars indicate the SD. The results are expressed as the mean ± SD of three independent experiments. **= $p < 0.01$, ***=$p < 0.001$ using the two-sided Student's *t*-test. Source data are provided as a Source Data file.

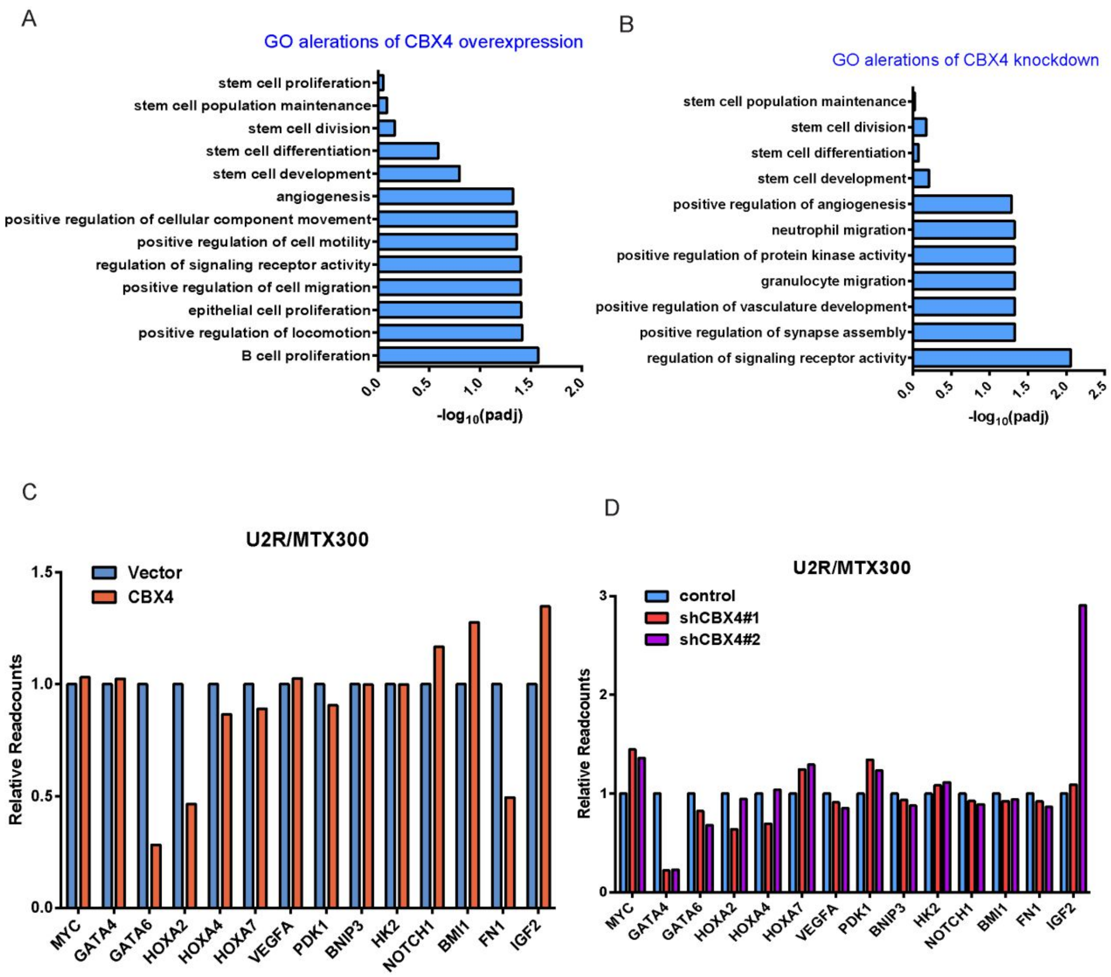

Supplementary Figure 5. RNA-seq analyses of both CBX4-overexpression and CBX4-knockdown in U2OS/MTX300 cells. (A and B) GO analysis of CBX4-overexpression **(A)** or CBX4-knockdown **(B)** in U2OS/MTX300 cells. **(C and D)** The analysis of other reported CBX4 target genes according to the RNA-seq data. Source data are provided as a Source Data file.

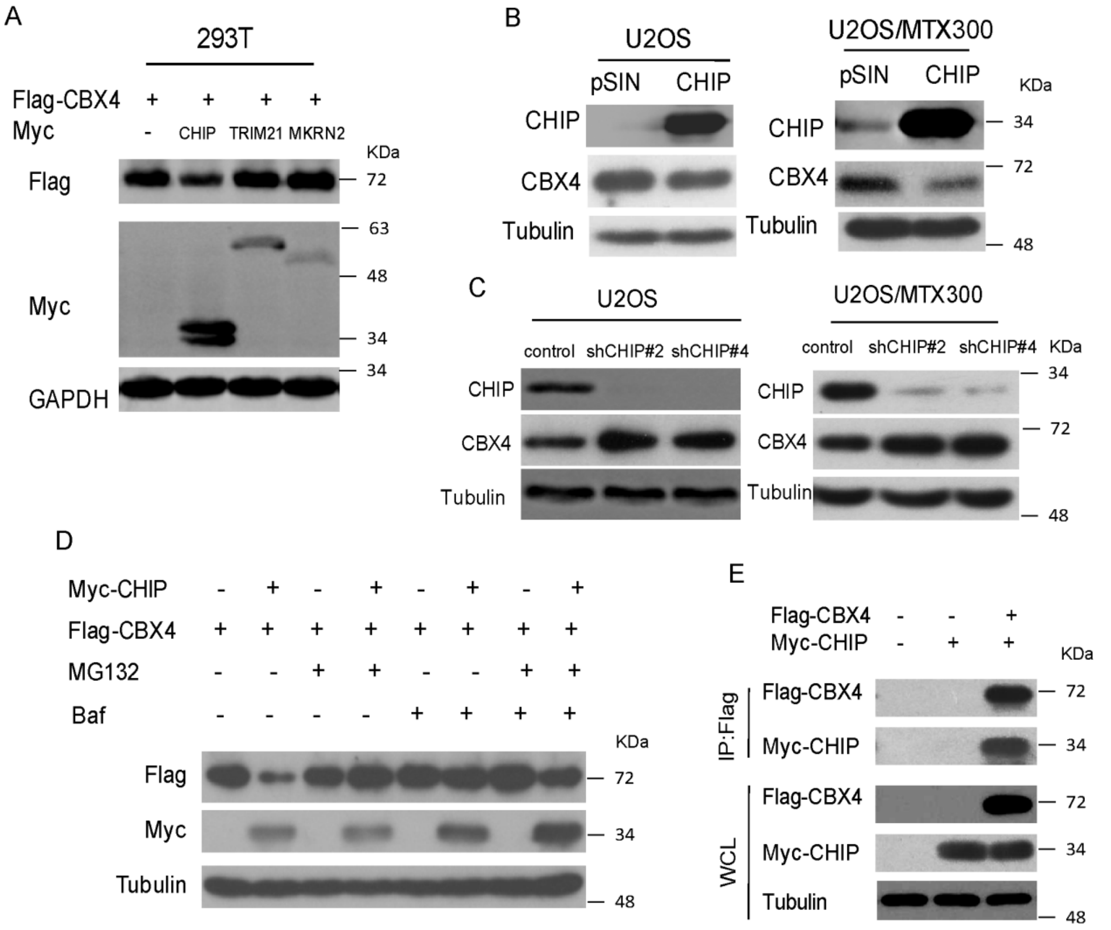

Supplementary Figure 6. CHIP is the E3 ligase responsible for the degradation of CBX4 by the proteasome / lysosome. **(A)** HEK293T cells transfected with the indicated plasmids 48 hours and then analyzed by Western blotting. **(B and C)** The indicated proteins were analyzed by Western blotting in the indicated stable cells. **(D)** HEK293T cells transfected with the indicated plasmids 40 hours were incubated with MG132 or bafilomycin (Baf) for 8 hours and then analyzed by Western blotting. **(E)** HEK293T cells transfected with the indicated plasmids were subjected to IP using Flag-agarose followed by Western blotting. These resluts are repeated of three independent experiments. Source data are provided as a Source Data file.

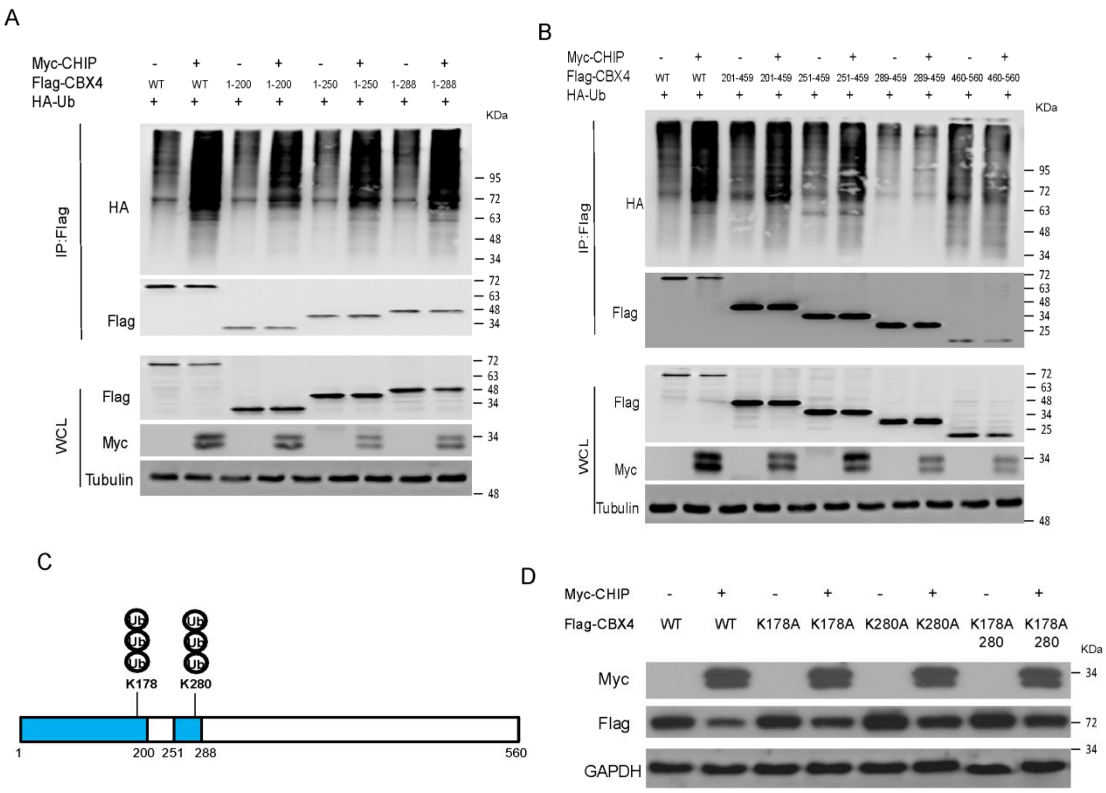

Supplementary Figure 7. Both K178 and K280 of CBX4 are key sites for ubiquitination by CHIP. (A, B) HEK293T cells transfected with the indicated plasmids were subjected to IP using Flag-agarose followed by Western blotting of three independent experiments. **(C)** Schematic illustration of the regions (1-200aa and 251-288aa) and sites (K178 and K280) of CBX4 ubiquitination. **(D)** HEK293T cells transfected with the indicated plasmids were subjected to Western blotting of three independent experiments. Source data are provided as a Source Data file.

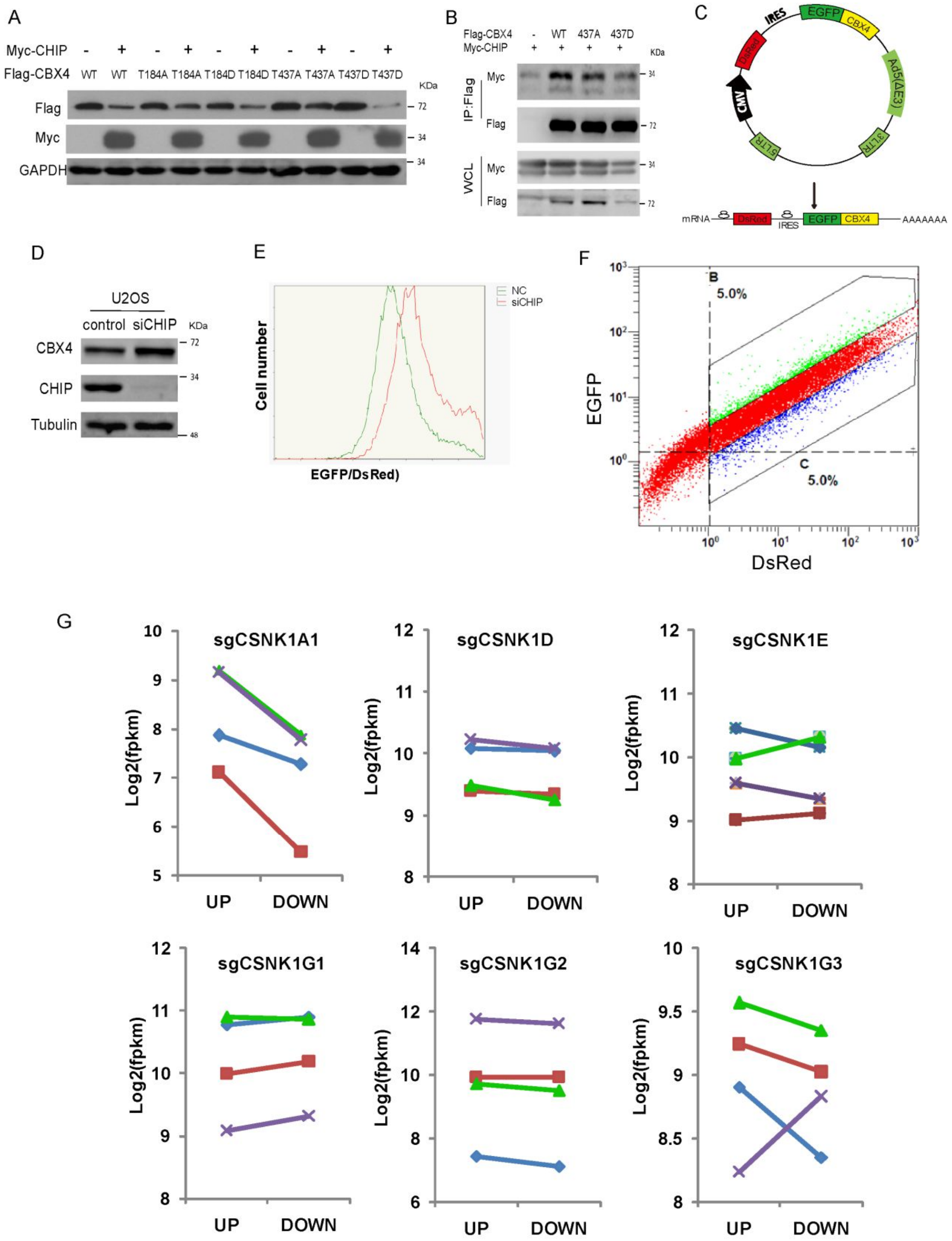
A
Myc-CHIP - + - + - + - + - +
Flag-CBX4 WT WT T184A T184A T184D T184D T437A T437A T437D T437D
KDa
Flag
Myc
GAPDH
72
34
34
B
Flag-CBX4 - WT 437A 437D
Myc-CHIP + + + +
KDa
IP:Flag
Myc
Flag
WCL
Myc
Flag
C
IRES
EGFP
CBX4
DsRed
CMV
Ad5(ΔE3)
5'LTR
3'LTR
mRNA
DsRed
IRES
EGFP
CBX4
AAAAAAA
D
U2OS
control siCHIP
KDa
CBX4
CHIP
Tubulin
72
34
48
E
NC
siCHIP
Cell number
EGFP/DsRed)
F
B
5.0%
C
5.0%
EGFP
DsRed
G
sgCSNK1A1
sgCSNK1D
sgCSNK1E
sgCSNK1G1
sgCSNK1G2
sgCSNK1G3
Log2(fpkm)
UP
DOWN

Supplementary Figure 8. CK1α down-regulates CBX4 protein stability. (**A**) HEK293T cells transfected with the indicated plasmids were subjected to Western blotting. (**B**) HEK293T cells transfected with the indicated plasmids were subjected to IP using Flag-agarose followed by Western blotting. **(C)** Schematic of the pAd-DsRed-IRES-EGFP-CBX4 (protein) reporter system. The DsRed-IRES-EGFP-CBX4 element was cloned into an adenovirus vector. (**D, E**) Stable U2OS cells infected with pAd-DsRed-IRESEGFP-CBX4 adenovirus were transfected with siCHIP for 48 hours and then analyzed by Western blotting **(D)** and flow cytometry **(E)**. These resluts are repeated of three independent experiments. **(F)** Gating strategy to sort cells of altered EGFP/DsRed ratios in U2OS transfected with the CRISPR-Cas9 kinase library. **(G)** The normalized read counts of individual sgRNAs targeting each CK1 isoforms in the high and low EGFP/DsRed ratio populations. Source data are provided as a Source Data file.

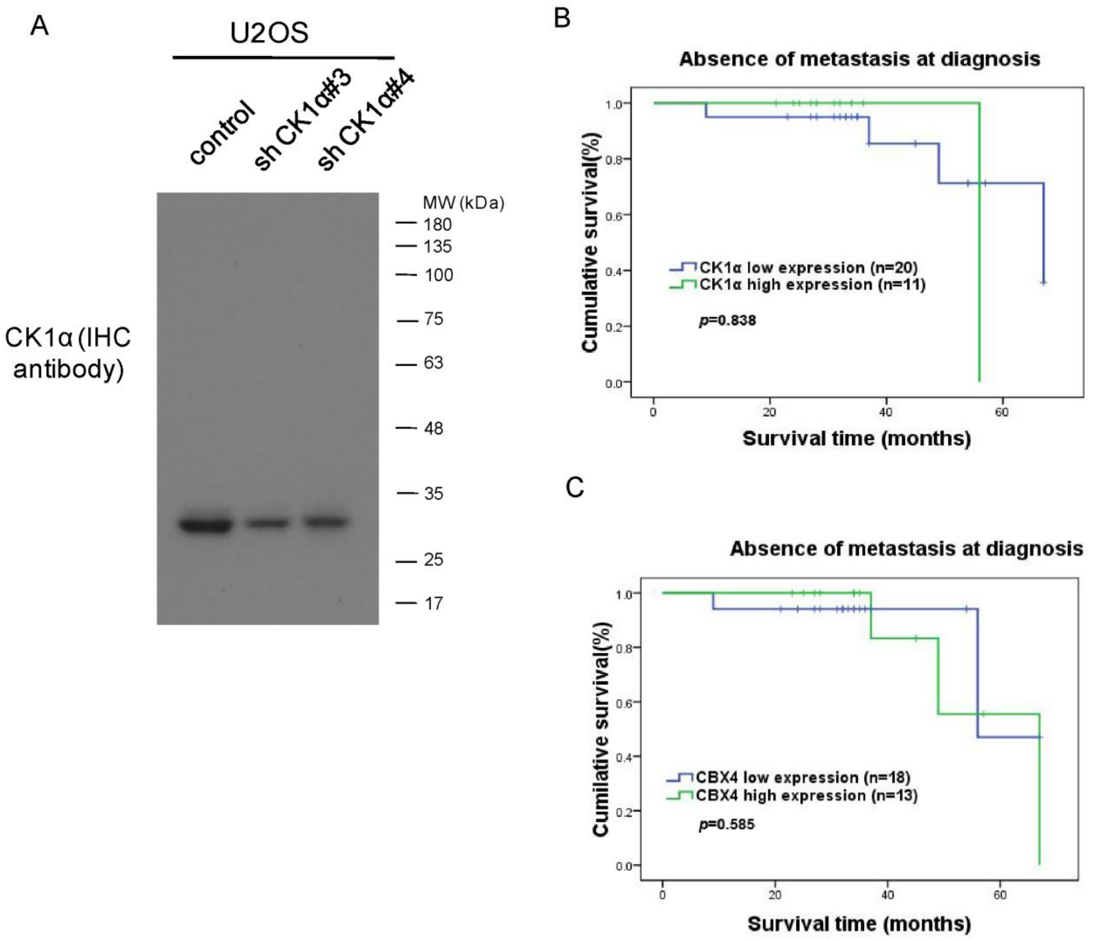

Supplementary Figure 9. The levels of CK1α and CBX4 were not associated with overall survival in patients without metastasis at diagnosis. (A) Whole cell lysates form U2OS cells stably expressing control or shRNA-CK1α were analyzed by Western blotting of three independent experiments. Source data are provided as a Source Data file. **(B, C)** Overall survival curves were generated based on the protein levels of CK1α **(B)** or CBX4 **(C)** by using Kaplan-Meier plots and compared with the log-rank test.

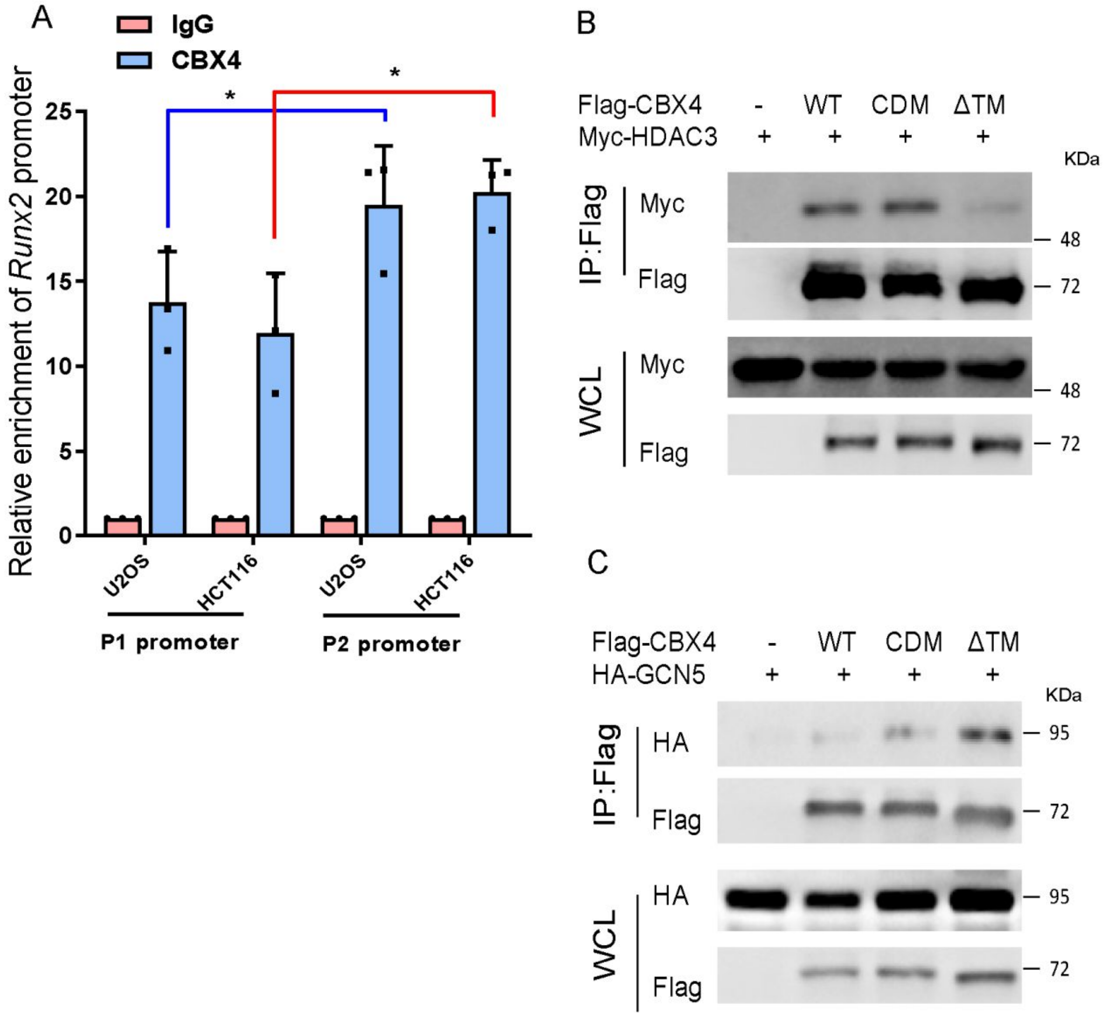

Supplementary Figure 10. (A) ChIP-qPCR analysis of the occupancies of CBX4 on the *Runx2* P1 or P2 promoter in the indicated stable cells. The bars indicate the SD. The results are expressed as the mean ± SD of three independent experiments. * $p< 0.05$, ** $p < 0.01$, ***$p<0.001$ using the two-sided Student's *t*-test. **(B and C)** HEK293T cells were co-transfected with the indicated plasmids for 48 h and then subjected to IP using anti-FLAG antibody followed by Western blotting analysis of three independent experiments. Source data are provided as a Source Data file.

Supplementary Table 1

The information on the 16 osteosarcoma samples and 4 normal samples for RNA-seq

Clinic number	Age	Gener	Tumor number	Tumor location	Metastasis
852003	16	male	T1	right distal femur	Yes
847031	17	male	T2	right proximal tibia	Yes
859912	10	male	T3	left distal tibia	No
847458	12	male	T4	right distal femur	No
858691	19	male	T5	right femur	Yes
858692	15	female	T6	right distal femur	Yes
844842	14	female	T7	right proximal tibia	No
829628	12	female	T8	right distal femur	Yes
865261	17	male	T9	right distal femur	Yes
834839	10	female	T10	left proximal tibia	Yes
1717647	8	female	T11	left distal femur	No
1730679	40	male	T12	right distal femur	No
1738179	15	male	T13	right proximal tibia	No
870456	18	male	T14	right distal femur	No
8119304	42	male	T15	left proximal femur	No
T1N0M0	20	male	T16	right tibia	No
Clinic number	Age	Gener	Normal number		
852003	16	male	N1		
847031	17	male	N2		
859912	10	male	N3		
847458	12	male	N4		

Supplementary Table 2

Interaction proteins with CBX4 identified by mass spectrometry

	**U2OS/MTX300**		**HEK293T**	
	**Protein**	**Number of Peptides**	**Protein**	**Number of Peptides**
PRC1 complex	Phc2	36	PCGF6	5
	Phc1	29	Phc2	5
	Ring1b	21	Phc3	2
	Bmi1	20	Bmi1	2
	Phc3	16	Ring1a	2
	Schm1	14	PCGF1	1
	PCGF2	14		
	CBX8	13		
	Ring1a	7		
	CBX2	6		
	PCGF6	2		
	CBX7	2		
Kinase or E3-ligase	PLK1	15	TRIM21	3
	CK2α	2	CK2α	2
	CK1α	1	CHIP	2
			MKRN2	1

Supplementary Table 3

CPLM1.0 – Compendium of Protein Lysine Modification

	Position	Peptide	Type
Lysine Modifivation	38	KWRGWSPKYNTWEPE	Ubiquitination
	114	LDLGAQGKGQGHQYE	Ubiquitination
	149	PPPGKSGKYYYQLNS	Acetylation
	178	LQYQGGHKEAPSPTC	Ubiquitination
	212	KGYLGAVKPLAGAAG	Ubiquitination
	249	TGNGIGGKMKIVKNK	Ubiquitination
	278	ENGMQAVKIKSGEVA	Ubiquitination
	280	GMQAVKIKSGEVAEG	Ubiquitination
	365	QPLQLTTKPDLLAWD	Ubiquitination
	494	PPSSLQVKPETPASA	Sumoylation

Supplementary Table 4

Phosphorylated sites of CBX4 identified by mass spectrometry

Protein	Position	Modified Sequence	Motifs
CBX4	184	YDLQYQGGHKEAPSPpTCPDLGAKSHPPD [EAPS(-7.99)PT(7.99)CPDLGAK]	CK2
CBX4	437	RLTARSISTPpTCLGGSPAAER [S(-13.04)IS(-12.43)T(-12.43)PT(11.57)CLGGS (-11.57)PAAER]	CK1(S/TxxS/T*)

Supplementary Table 5

Information about p-437 antibody of CBX4

Protein	Position	Modification	Immunogen	Clone Number
CBX4	T437	phosphorylation	(aa431-442)：<br>C-RSISTP(pT)CLGGS	Rb6209

Supplementary Materials

Plasmid construction

CCGGCGTGATCGTGATGAGCAAATACTCGAGTATTTGCTCATCACGATCACG TTTTTG for shCBX4#1,

CCGGGAGTGGAGTATCTGGTGAAATCTCGAGATTTCACCAGATACTCCACT CTTTTTG for shCBX4#2,

CCGGGCAAGCTCTATAAGATTCTTCCTCGAGGAAGAATCTTATAGAGCTTGC TTTTTG for shCK1α#3,

CCGGGCAGAATTTGCGATGTACTTACTCGAGTAAGTACATCGCAAATTCTGC TTTTTG for shCK1α#4,

CCGGGTGGTCCTATGACCAGTCTCTCGAGAGACTGGTCATAGGACCACTTT TTG for shRunx2#2,

CCGGTGCACTATCCAGCCACCTTCTCGAGAAGGTGGCTGGATAGTGCATT TTTG for shRunx2#3,

CCGGAGAGGAAGAAGCGAGACATCTCGAGATGTCTCGCTTCTTCCTCTTTT TTG for shCHIP#2,

CCGGTGCCGCCACTATCTGTGTACTCGAGTACACAGATAGTGGCGGCATTTT TG for shCHIP#4

RNA extraction and qRT-PCR

The primers used to amplify the indicated genes are as follows.

	**qRT-PCR Primers**	
**Gene**	**Forward Sequence(5′-3′)**	**Reverse sequence(5′-3′)**
Runx2	CCGCCTCAGTGATTTAGGGC	GGGTCTGTAATCTGACTCTGTCC
Ring1b	ACCCAAACTTTGATGCACTCA	TTGTGCTTGTTGATCCTGGCT
HDAC1	CGCCCTCACAAAGCCAATG	CTGCTTGCTGTACTCCGACA
HDAC2	GAGCTGTGAAGTTAAACCGACA	ACCGTCATTACACGATCTGTTG
HDAC3	TCTGGCTTCTGCTATGTCAACG	CCCGGTCAGTGAGGTAGAAAG
GAPDH	ACAGTCAGCCGCATCTTCTT	GACAAGCTTCCCGTTCTCAG

The luciferase reporter assay

The primers used for cloning the indicated promoters are as follows.

	**Primers for promoter clone**	
**Gene**	**Forward Sequence(5′-3′)**	**Reverse sequence(5′-3′)**
Runx2 P2	TTTCTCTATCGATAGGTACCAAACCAGACAGGGAGGGTCGTG	GATCGCAGATCTCGAGGCTGAGGAGGTGAGAAATC

The chromatin immunoprecipitation (ChIP) assay

The primers for the indicated promoters are as follows.

	**ChIP-qPCR Primers**	
**Gene**	**Forward Sequence(5′-3′)**	**Reverse sequence(5′-3′)**
Runx2 P2	TGGACTGCTGAACCCACAC	TGAGTTTGCAGCTTGGAATG
Runx2 P1	CTTCAAACTAGGCATGAGATAATGG	CACATTCTGGGGTCTGCTTCATATT
p16	CGGCTGGGAGCAGGGAGGC	GAATGTGGCACCCCTGAGTCGC

Western blotting and Co-inmmunoprecipatation (Co-IP)

The antibodies used in this work are as follows.

**Antibody target**	**Vendor**	**Catalog No.**
CBX4	Bethyl	A302-355A
Runx2	CST	8486s
CK1α	Abcam	ab108296
GCN5	Abcam	ab217876
CHIP	CST	2080S
HA	CST	3724S
Tubulin	Bioworld	AP0064
V5	invitrogen	46-0705
RNA polymerase II	Millipore	05-623B
H3K27ac	CST	8173S
HA-agrose beads	sigma	A2095
Flag	CST	2368S
Flag-agrose beads	Sigma	A4596
Myc	CST	2272
GAPDH	CST	2118
Myc-agrose beads	CMCTAG	AT0080
CBX4(IHC)	Sigma	HPA008228
β-catenin	proteintech	51067-2-AP
p-T437		Rb6209
