# Supplementary material for: Targeting the CK1α/CBX4 axis for metastasis in osteosarcoma
Source: Nat Commun. 2020 Feb 28;11:1141. doi: 10.1038/s41467-020-14870-4 (PMC7048933; doi:10.1038/s41467-020-14870-4)
Supplement: Supplementary file 2 — Description of Additional Supplementary Files [file 41467_2020_14870_MOESM2_ESM.docx]

**Description of Additional Supplementary Files**

File name: Supplementary Data 1

Description: The clinical information of 55 osteosarcoma patients.
